# Advances in Precision Oncology: From Molecular Profiling to Regulatory-Approved Targeted Therapies

**DOI:** 10.3390/cancers17213500

**Published:** 2025-10-30

**Authors:** Petar Brlek, Vedrana Škaro, Nenad Hrvatin, Luka Bulić, Ana Petrović, Petar Projić, Martina Smolić, Parth Shah, Dragan Primorac

**Affiliations:** 1 St. Catherine Specialty Hospital, 10000 Zagreb, Croatialuka.bulic@svkatarina.hr (L.B.);; 2 International Center for Applied Biological Research, 10000 Zagreb, Croatia; 3 Department of Molecular Biology, Faculty of Science, University of Zagreb, 10000 Zagreb, Croatia; 4 School of Medicine, Josip Juraj Strossmayer University of Osijek, 31000 Osijek, Croatia; 5 Department of Pediatrics, Clinical Hospital Center Rijeka, 51000 Rijeka, Croatia; 6 Faculty of Dental Medicine and Health, Josip Juraj Strossmayer University of Osijek, 31000 Osijek, Croatia; 7 Dartmouth Health, Lebanon, NH 03766, USA; 8 Eberly College of Science, The Pennsylvania State University, State College, PA 16802, USA; 9 School of Medicine, University of Split, 21000 Split, Croatia; 10 The Henry C. Lee College of Criminal Justice and Forensic Sciences, University of New Haven, New Haven, CT 06516, USA; 11 Sana Kliniken Oberfranken, 96450 Coburg, Germany; 12 School of Medicine, University of Rijeka, 51000 Rijeka, Croatia; 13 School of Medicine, University of Mostar, 88000 Mostar, Bosnia and Herzegovina; 14 National Forensic Sciences University, Gandhinagar 382007, India

**Keywords:** precision oncology, whole-genome sequencing, RNA sequencing, immunotherapy, personalized medicine, artificial intelligence

## Abstract

**Simple Summary:**

Cancer develops through a complex series of genetic and molecular changes. Modern sequencing technologies now allow scientists and physicians to study these changes in great detail, helping them understand why each person’s cancer behaves differently. By examining the entire genome, exome, or transcriptome of a tumor, researchers can uncover genetic alterations that guide personalized treatment decisions. This article explains how these sequencing methods, together with data analysis tools and artificial intelligence, help identify the best targeted and immune-based therapies for different cancer types. By linking genetic findings to approved drugs, the study aims to make precision cancer care more effective and accessible for patients while also guiding future research directions in oncology.

**Abstract:**

The rapid evolution of sequencing technologies has profoundly advanced precision oncology. Whole-exome sequencing (WES), whole-genome sequencing (WGS), and whole-transcriptome sequencing (RNA-Seq) enable comprehensive characterization of tumor biology by detecting actionable mutations, gene fusions, splice variants, copy number alterations, and pathway dysregulation. These approaches also provide critical insights into biomarkers such as homologous recombination deficiency (HRD), tumor mutational burden (TMB), and microsatellite instability (MSI), which are increasingly essential for guiding therapeutic decisions. Importantly, comprehensive genomic profiling not only refines patient stratification for targeted therapies but also sheds light on tumor–immune interactions and the tumor microenvironment, paving the way for more effective immunotherapeutic combinations. WGS is considered the gold standard for detecting germline mutations and complex structural variants, while WES remains central for detecting somatic driver mutations that guide targeted therapies. RNA-Seq complements these methods by capturing gene expression dynamics, identifying clinically relevant fusions, and revealing mechanisms of resistance. Together with advances in bioinformatics and artificial intelligence, these tools translate molecular data into actionable strategies for patient care. This review integrates insights from WGS, WES, and RNA-Seq with an overview of FDA- and EMA-approved targeted therapies, organized by tumor type, and highlights the molecular signaling pathways that drive cancer development and treatment. By bridging genomic profiling with regulatory-approved therapies, we outline current advances and future perspectives in delivering personalized cancer care.

## 1. Introduction

Cancer continues to represent a major global health burden and remains among the foremost causes of morbidity and mortality. Despite incremental advances achieved through surgical resection, cytotoxic chemotherapy, and radiotherapy, clinical outcomes for patients with advanced-stage malignancies are frequently suboptimal [1,2]. The fundamental limitation of conventional therapeutic modalities lies in their non-selective mechanisms of action, which not only attenuate efficacy but also confer substantial systemic toxicity. These constraints have underscored the imperative for therapeutic paradigms capable of delivering heightened specificity, thereby aligning treatment more closely with the molecular underpinnings of individual neoplasms [2].

Within this context, the advent of precision oncology has precipitated a paradigm shift in contemporary cancer care. The integration of molecular diagnostics into clinical decision-making has enabled a transition from histology- or site-based treatment algorithms toward approaches informed by the genetic, epigenetic, and transcriptomic aberrations that drive tumorigenesis [3]. Central to this shift is the deployment of targeted therapies—agents designed to inhibit discrete oncogenic drivers or signaling pathways essential for tumor cell proliferation and survival. Clinical adoption of such agents has yielded demonstrable improvements in progression-free and overall survival across multiple tumor types, including non-small cell lung carcinoma, breast carcinoma, and colorectal carcinoma [4,5,6].

This therapeutic progress has been facilitated by rapid innovations in high-throughput sequencing technologies. Whole-exome sequencing (WES), whole-genome sequencing (WGS), and RNA sequencing (RNA-Seq) permit comprehensive interrogation of tumor genomes and transcriptomes, thereby enabling the identification of clinically actionable genomic alterations, structural rearrangements, splice variants, and pathway dysregulation [7,8]. In addition, characterization of predictive biomarkers—including homologous recombination deficiency (HRD), microsatellite instability (MSI) and tumor mutational burden (TMB)—has refined patient stratification and expanded opportunities for individualized treatment selection [9,10].

Concomitantly, advances in computational bioinformatics and artificial intelligence have augmented the interpretation of multidimensional molecular datasets, accelerating the identification of novel therapeutic targets and illuminating mechanisms of primary and acquired resistance [11]. Regulatory agencies such as the U.S. Food and Drug Administration (FDA) and the European Medicines Agency (EMA) have responded to these scientific developments by approving an expanding repertoire of targeted agents, encompassing tyrosine kinase inhibitors, monoclonal antibodies, angiogenesis inhibitors, and other classes of precision therapeutics [12,13].

The objective of this review is to provide a rigorous and clinically oriented synthesis of current knowledge on targeted therapies, with particular emphasis on the translational application of molecular profiling to therapeutic decision-making. By consolidating insights derived from next-generation sequencing technologies with the evolving landscape of FDA- and EMA-approved targeted agents, this review seeks to delineate the interface between genomic science and personalized oncology practice and to outline the future trajectory of precision-based cancer therapeutics.

## 2. Modern Diagnostic Tools in Precision Oncology

### 2.1. Tumor Molecular Profiling Through Sequencing

Molecular profiling constitutes the foundation of precision oncology, enabling the systematic identification of clinically actionable genomic and transcriptomic alterations that inform therapeutic decision-making. While histopathology and immunohistochemistry remain indispensable for cancer diagnosis and classification, the advent of next-generation sequencing (NGS) technologies has revolutionized the scope and depth of tumor characterization [14,15]. Approaches such as whole-exome sequencing (WES), whole-genome sequencing (WGS), and whole-transcriptome sequencing (RNA-Seq) provide multidimensional insights into the molecular architecture of malignancies, revealing somatic mutations, structural rearrangements, dysregulated signaling networks, and novel biomarkers with direct clinical relevance [7,16].

### 2.2. Whole Genome (WGS) and Whole Exome Sequencing (WES)

Whole-genome sequencing interrogates the entire human genome (~3.2 billion base pairs), encompassing both coding and non-coding regions [17,18]. This comprehensive approach permits unbiased detection of single nucleotide variants (SNVs), insertions and deletions (indels), copy number variations (CNVs), structural rearrangements, mutational signatures, and epigenetic modifications. In contrast, whole-exome sequencing targets the ~1–2% of the genome that encodes proteins, thereby enriching for regions most likely to harbor driver mutations, while simultaneously reducing sequencing costs and computational complexity relative to WGS [7,19]. Both methodologies are typically performed in a tumor–normal matched fashion, facilitating robust discrimination between somatic alterations and germline variants.

From a clinical standpoint, WGS is regarded as the most comprehensive platform for detecting germline variants associated with hereditary cancer predisposition syndromes, such as Lynch syndrome, Li-Fraumeni syndrome, and hereditary breast and ovarian cancer [20,21,22]. By spanning coding and non-coding regions, WGS also excels in identifying large-scale structural variants—including chromosomal translocations, duplications, deletions, and complex rearrangements—that may elude targeted sequencing panels. Conversely, gene panels remain the predominant method for the detection of somatic driver mutations in tumor tissue, alongside WES in some institutions, given their high coverage of exonic regions where the majority of actionable alterations reside (e.g., *EGFR*, *BRAF*, *PIK3CA*, *KRAS*, *BRCA1/2*) [23,24]. These data directly inform the selection of targeted therapies, elucidate mechanisms of acquired resistance, and contribute to prognostic and predictive stratification [25].

Importantly, both WGS and WES are increasingly recognized by regulatory authorities and payers as integral to comprehensive genomic profiling, serving as prerequisites for access to genotype-directed therapies and eligibility for biomarker-driven clinical trials. Their complementary utilities—WGS in delineating hereditary predisposition and complex structural variation, WES in detecting high-yield somatic driver mutations—underscore their indispensable roles in contemporary precision oncology [7,26].

### 2.3. Whole-Transcriptome Sequencing (RNA-Seq)

Whereas WGS and WES delineate the static genomic blueprint, transcriptomic analysis provides a dynamic representation of gene expression within the tumor and its microenvironment. Transcriptome sequencing enables the identification of oncogenic gene fusions (e.g., *ALK*, *ROS1*, *NTRK, BCR-ABL1, ETV6-NTRK3*), alternative splicing events, allele-specific expression patterns, and quantitative transcript levels, thereby capturing both functional and regulatory dimensions of tumor biology. RNA-Seq is particularly powerful for identifying these fusions because it captures the actual transcripts produced, allowing for precise mapping of fusion breakpoints and expression levels. Beyond simple cataloging, RNA-Seq elucidates pathway activation states, tumor–immune interactions, and transcriptional adaptations underlying therapeutic resistance [27,28].

Clinically, RNA-Seq proves particularly valuable in scenarios where diagnostic ambiguity persists. In sarcomas or poorly differentiated carcinomas, for example, the detection of pathognomonic fusion events via RNA-Seq can establish a definitive molecular diagnosis. Moreover, RNA-Seq has demonstrated superiority in uncovering clinically actionable fusions (e.g., *RET*, *NRG1*) that may evade detection by DNA-based sequencing approaches due to large intronic regions or structural complexity. By providing functional context and complementing DNA-based genomic profiling, RNA-Seq substantially augments the capacity to identify therapeutically relevant alterations and expand patient eligibility for targeted interventions [27,29].

### 2.4. Complementary Molecular Diagnostic Techniques in Precision Oncology

Despite the widespread adoption of sequencing technologies, fluorescence in situ hybridization (FISH) and immunohistochemistry (IHC) remain integral components of routine oncological diagnostics. These assays are standardized, reproducible, and directly linked to treatment selection.

FISH enables precise detection of gene amplifications, deletions, and rearrangements. In breast and gastric cancers, confirmation of HER2 amplification by FISH remains the reference standard for selecting patients for trastuzumab and other HER2-directed therapies. In non-small cell lung cancer (NSCLC), identification of ALK, ROS1, or RET fusions provides access to tyrosine kinase inhibitors with substantial clinical benefit [30,31]. Demonstration of the BCR-ABL translocation continues to underpin therapeutic stratification in chronic myeloid leukemia through the use of imatinib and subsequent generations of TKIs. In neuro-oncology, detection of the 1p/19q codeletion is diagnostic of oligodendroglioma, distinguishing it from astrocytic gliomas, and predicts enhanced responsiveness to combined chemotherapy and radiotherapy, with clear prognostic implications [32,33,34].

IHC remains the cornerstone for assessing hormone receptor (ER, PR) and HER2 status in breast cancer, guiding endocrine and HER2-targeted therapies [35]. Evaluation of PD-L1 expression by IHC serves as a predictive biomarker across multiple tumor types for the use of immune checkpoint inhibitors targeting PD-1/PD-L1 [36]. Testing of mismatch repair (MMR) proteins identifies tumors with microsatellite instability (MSI), indicating possible Lynch syndrome and predicting benefit from pembrolizumab [37,38]. Assessment of the Ki-67 proliferation index has established value in routine practice: in neuroendocrine tumors it determines histological grade (G1–G3) and informs treatment strategy, while in lymphomas it provides prognostic information on tumor aggressiveness and influences therapeutic intensity [39,40].

Although often regarded as conventional methods, FISH and IHC continue to represent the backbone of biomarker testing in oncology. They provide rapid, cost-effective, and clinically validated results that are essential for integrating molecular findings into therapeutic decision-making. Importantly, they serve as a practical bridge between histopathological evaluation and next-generation sequencing, ensuring that patient management is aligned with the molecular architecture of each tumor.

In modern molecular oncology, clinical decision-making increasingly relies on an integrated multi-omics framework that concurrently interrogates germline and somatic genomics (SNVs, CNVs, fusions), epigenomics (DNA methylation, chromatin accessibility), transcriptomics, proteomics, glycomics, and metabolomics/lipidomics. This multi-omics strategy is often complemented by single-cell and spatial assays and computational pathology to quantify pathway activity and immune modulation, predict drug efficacy and toxicity (pharmacogenomics), and more precisely direct biomarker-driven targeted therapies.

### 2.5. Pharmacogenomics in Oncology

In addition to the molecular methods outlined above—particularly whole-genome sequencing (WGS)—it is possible to define a patient’s pharmacogenetic profile that informs drug metabolism and the risk of adverse effects from standard chemotherapeutics. While this is possible through gene panels and WES, WGS provides the optimal way to assess SVs and CNVs in these genes. In clinical practice, genotyping of the gene encoding dihydropyrimidine dehydrogenase (DPYD), which metabolizes capecitabine and 5-fluorouracil, is already implemented with dosing recommendations guided by Clinical Pharmacogenetics Implementation Consortium (CPIC) guidelines. It is also important to highlight the clinical relevance of *UGT1A1* genotyping for irinotecan dose selection. In addition, state-of-the-art clinical practice recognizes the value of *CYP2D6* genotyping to estimate tamoxifen metabolic capacity. Integrating pharmacogenomics into routine clinical practice can reduce the risk of improper dosing and enhance treatment safety [41,42,43].

### 2.6. Artificial Intelligence in Precision Oncology

Artificial intelligence (AI) has emerged as a pivotal adjunct in the evolution of precision oncology, offering powerful computational frameworks for variant classification, biomarker discovery, therapeutic response prediction, and individualized treatment optimization. By integrating multidimensional datasets—including genomic, transcriptomic, epigenomic, proteomic, and clinical parameters—AI-based models can discern clinically relevant patterns with a level of precision and scalability unattainable through conventional statistical methodologies [44,45]. In particular, deep learning architectures have demonstrated the ability to identify complex molecular signatures and latent features that are imperceptible to human interpretation or traditional bioinformatics pipelines. Such applications extend beyond diagnostic refinement to include prognostic stratification, therapeutic target identification, and resistance mechanism elucidation, thereby positioning AI as a catalyst for molecularly informed decision-making [46].

A salient example of clinical utility is found in the management of cancers of unknown primary (CUP), a biologically heterogeneous group of metastatic malignancies in which the anatomical site of origin remains elusive despite exhaustive clinical, radiologic, and histopathologic assessment. Diagnostic uncertainty in CUP severely constrains therapeutic choices and contributes to unfavorable clinical outcomes [47]. Recent investigations have demonstrated that AI-driven analytic platforms, when applied to comprehensive molecular profiling datasets, can accurately predict tissue of origin and, in some instances, delineate actionable genomic alterations [48]. These capabilities hold substantial promise for mitigating diagnostic ambiguity, expanding access to targeted therapies, and ultimately reshaping the therapeutic landscape for patients with CUP.

## 3. Molecular Signaling Pathways in Cancer

The initiation and progression of malignancy arise from a multifaceted convergence of oncogenic activation, tumor suppressor gene inactivation, aberrant signaling network dynamics, and immune evasion strategies. Genetic alterations that directly contribute to the initiation and progression of cancer, also called oncogenic driver mutations, typically occur in genes that regulate cell growth, division, and survival, and they give cancer cells a growth advantage over normal cells (Table 1).

More precisely MAPK/ERK pathway—controls cell proliferation, differentiation, cell survival and apoptosis, gene expression regulation, cell cycle progression and migration and invasion, PI3K/AKT/mTOR pathway—regulates cell survival, metabolism, and growth, WNT/β-catenin pathway—involved in stem cell maintenance and differentiation, JAK/STAT pathway—transmits signals from cytokines and growth factors, while DNA damage response pathways—involve BRCA1/2, ATM, and TP53. In parallel, the functional loss of tumor suppressor genes abrogates genomic integrity checkpoints and cell-cycle regulation, eliminating critical safeguards against malignant transformation. Concurrently, neoplastic cells exploit immune checkpoint signaling, most notably through PD-1/PD-L1 and CTLA-4 axes, to subvert host immune surveillance and establish an immunosuppressive tumor microenvironment [49]. Collectively, these perturbations create a permissive molecular milieu conducive to tumor initiation, clonal evolution, therapy resistance, and metastatic dissemination [50].

From a translational standpoint, recurrent alterations within oncogenic and tumor suppressor networks have provided the mechanistic rationale for the development of both targeted therapies and immunomodulatory agents that have reshaped the therapeutic armamentarium in oncology. Mutations in canonical oncogenes such as *BRAF*, *KRAS*, *PIK3CA*, and *ALK* function as dominant molecular drivers, defining biologically distinct tumor subsets characterized by specific therapeutic liabilities [34,51]. Conversely, loss-of-function alterations in pivotal tumor suppressors—including *TP53*, *RB1*, and *PTEN*—remove essential regulatory nodes governing genomic stability, DNA damage repair, and programmed cell death [34,52]. The interplay between oncogene-driven signaling hyperactivation and tumor suppressor loss constitutes the molecular substrate of malignant transformation, underscoring the clinical imperative for comprehensive genomic profiling to inform both prognosis and the rational selection of genotype-directed therapies.

### 3.1. Key Pathways in Oncogenesis

#### 3.1.1. MAPK/ERK Pathway

**Figure 1:**
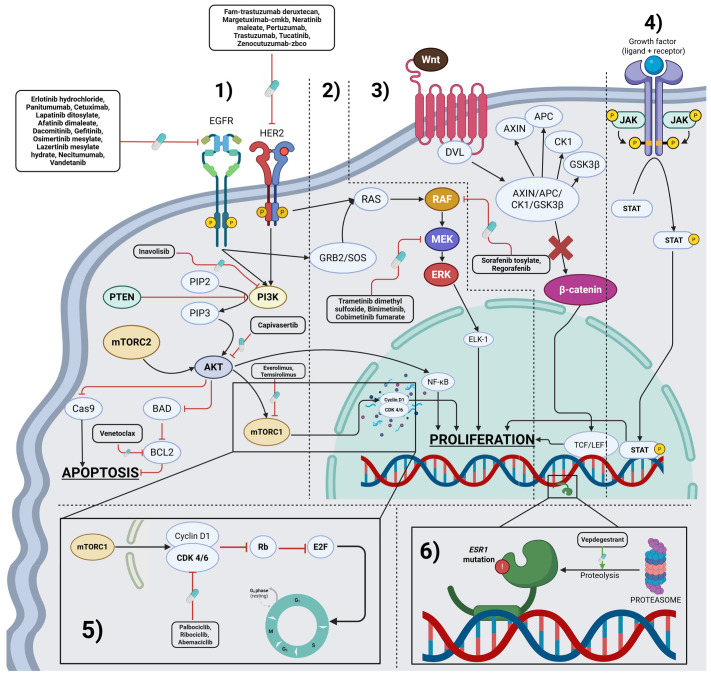
Major oncogenic signaling networks in cancer. The PI3K/AKT/mTOR cascade (1) promotes growth and survival, while the MAPK/ERK pathway (2) drives proliferation via RAS–RAF–MEK–ERK activation. The Wnt/β-catenin pathway (3) regulates cell fate and proliferation. Additionally, the JAK-STAT pathway (4) promotes proliferation via phosphorylation. Upstream, the EGFR/HER2 receptor axis functions as a central signaling hub and therapeutic target, with multiple inhibitors directed against its activation. CDK 4/6 inhibitors (5) can halt the cell cycle directly, by allowing Rb to exact its inhibitory effect on E2F. In the case of *ESR1* mutations (6), targeted therapy can stimulate proteolysis of the abnormal protein, halting aberrant proliferation stimulation (created with Biorender.com accessed on 20 September 2025).

The RAS-RAF-MEK-ERK pathway, also known as the MAPK/ERK signaling cascade, is a critical intracellular signaling pathway that regulates several fundamental cellular processes. Key components of the pathway are: RAS (a small GTPase that act as a molecular switch), RAF (a serine/threonine kinase activated by RAS), MEK (MAPK/ERK kinase) which is dual-specificity kinase that phosphorylates ERK, which is the last component of this pathway and as extracellular signal-regulated kinase-the final effector that enters the nucleus to regulate gene expression. The MAPK/ERK signaling cascade is one of the most frequently dysregulated pathways in human malignancies [53]. Activating mutations in *BRAF* (gene coding B-Raf protein)—most notably the V600E substitution (most commonly found in melanoma, thyroid cancer, colorectal cancer, and non-small cell lung carcinoma)—or in *KRAS* confer constitutive downstream signaling, driving uncontrolled cellular proliferation, survival, and metastatic potential. Therapeutic targeting of this pathway has yielded major advances in precision oncology. Selective *BRAF* inhibitors (dabrafenib, vemurafenib, encorafenib) and *MEK* inhibitors (trametinib, cobimetinib, binimetinib) have demonstrated robust clinical activity, particularly in melanoma, non-small cell lung carcinoma (NSCLC), and colorectal carcinoma harboring MAPK pathway alterations [54]. Despite the emergence of acquired resistance mechanisms, combinatorial and next-generation inhibitors continue to expand therapeutic efficacy. A schematic representation of the pathway, with therapeutic inhibitors annotated at their molecular targets, is provided in Figure 1.

#### 3.1.2. PI3K/AKT/mTOR Pathway

The phosphoinositide 3-kinase (PI3K)/AKT/mammalian target of rapamycin (mTOR) axis orchestrates key processes in cellular metabolism, growth, and survival, and constitutes a central oncogenic driver across multiple tumor types. Dysregulation commonly results from activating *PIK3CA* mutations, loss of function of the tumor suppressor *PTEN*, or amplification of receptor tyrosine kinases [34]. Pharmacologic inhibition of this pathway has been clinically validated with selective PI3K inhibitors (alpelisib, inavolisib), AKT inhibitors (capivasertib), and mTOR inhibitors (everolimus, temsirolimus) [55]. Importantly, aberrant PI3K/AKT/mTOR signaling is a well-recognized mediator of resistance to upstream targeted therapies, such as HER2- and EGFR-directed agents, underscoring its therapeutic relevance and justifying continued clinical development of novel inhibitors and rational combination strategies [56,57]. A schematic depiction of the pathway, highlighting approved and investigational inhibitors at their molecular nodes, is shown in Figure 1.

#### 3.1.3. Wnt/β-Catenin Pathway

The WNT/β-catenin signaling cascade plays a fundamental role in embryonic development and tissue homeostasis, and its aberrant activation constitutes a hallmark of several malignancies. Pathway dysregulation most frequently arises through truncating mutations in *APC* or activating mutations in *CTNNB1*, which promote nuclear β-catenin accumulation, transcriptional activation of oncogenic targets, and epithelial–mesenchymal transition (EMT) [58,59,60]. Aberrant WNT signaling is most common in colorectal carcinoma, but recent studies have shown that dysregulation of this pathway is also frequently implicated in other tumor types, including astrocytomas and glioblastomas [60,61]. Despite its biological importance, pharmacologic targeting of this pathway remains a major therapeutic challenge due to pathway complexity and toxicity concerns. Current investigational strategies include transcriptional modulators, porcupine inhibitors, and synthetic lethal approaches designed to indirectly suppress WNT-driven oncogenesis [62]. A schematic overview of this pathway, including emerging therapeutic strategies, is presented in Figure 1.

#### 3.1.4. JAK–STAT Pathway

The JAK–STAT signaling pathway is a streamlined mechanism that conveys signals from extracellular molecules directly to the nucleus, influencing gene expression. It is primarily activated by cytokines and growth factors, making it essential for immune regulation, hematopoiesis, and inflammatory responses. Signal initiation begins when a ligand binds to its receptor on the cell surface. This interaction activates Janus kinases (JAKs), which are non-receptor tyrosine kinases associated with the intracellular domain of the receptor [63].

Once activated, JAKs phosphorylate specific tyrosine residues on the receptor, creating docking sites for STAT proteins (Signal Transducers and Activators of Transcription). STATs bind to these phosphorylated sites and are themselves phosphorylated by JAKs. Phosphorylated STATs then dimerize and translocate into the nucleus, where they bind to DNA and regulate transcription of genes involved in cell proliferation, differentiation, apoptosis, and immune [63]. A schematic overview of this pathway is presented in Figure 1.

Aberrant activation of the JAK–STAT pathway has been implicated in various diseases, including autoimmune disorders, hematologic malignancies, and solid tumors. Therapeutic inhibition of JAKs (e.g., ruxolitinib, tofacitinib) has become a successful strategy in treating conditions such as myelofibrosis, rheumatoid arthritis, and ulcerative colitis [63,64]

### 3.2. Hormonal Pathways

**Figure 2:**
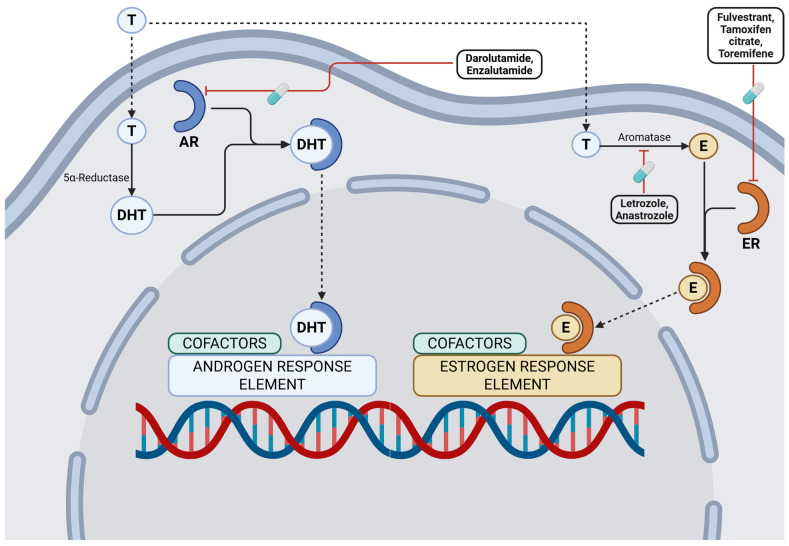
Hormonal signaling in cancer. AR-Androgen receptor activation by DHT and estrogen receptor activation by estrogen regulate gene expression through specific response elements. Therapeutic inhibitors of AR, aromatase, and ER-Estrogen receptors are indicated at key regulatory nodes. T—testosterone; DHT—dihydrotestosterone; E—estrogen (created with Biorender.com accessed on 20 September 2025).

Hormone receptor-mediated signaling represents a critical oncogenic axis in breast and prostate cancer biology. Estrogen receptor (ER) and progesterone receptor (PR) pathways drive the majority of breast cancers, while androgen receptor (AR) signaling underpins the growth and progression of prostate carcinomas [65]. Therapeutic strategies targeting these pathways have transformed clinical outcomes and remain among the most successful paradigms of precision oncology. Established interventions include ER antagonists (tamoxifen, enzalutamide), aromatase inhibitors (letrozole, anastrozole), androgen biosynthesis inhibitors (abiraterone), and selective ER degraders (fulvestrant, elacestrant) [66,67,68]. The development of next-generation hormonal agents continues to extend survival and delay resistance in hormone-driven malignancies. An illustration of the principal hormonal pathways and the molecular targets of corresponding agents is provided in Figure 2.

### 3.3. Immune Checkpoint Pathways

Immune checkpoint signaling constitutes a fundamental mechanism by which tumors evade host immunosurveillance, and therapeutic inhibition of these inhibitory pathways has redefined the landscape of cancer therapy [49]. The two most extensively characterized and clinically validated checkpoints are the programmed death 1/programmed death ligand 1 (PD-1/PD-L1) axis and cytotoxic T-lymphocyte-associated protein 4 (CTLA-4) [69]. Blockade of these inhibitory receptors restores effector T-cell activity within the tumor microenvironment, resulting in durable antitumor responses across multiple malignancies.

#### 3.3.1. PD-1/PD-L1 Pathway

The programmed cell death protein 1 (PD-1) is an inhibitory receptor expressed on activated T cells, B cells, and natural killer (NK) cells, and functions as a critical negative regulator of adaptive immunity [70]. Engagement of PD-1 with its principal ligands, PD-L1 and PD-L2—transmits a negative signal that reduces T cell proliferation, cytokine production, and cytotoxic activity. This interaction is crucial for maintaining immune tolerance and preventing autoimmunity. However, in the tumor microenvironment, cancer cells often upregulate PD-L1 to evade immune surveillance by suppressing antitumor T cell responses [71].

**Figure 3:**
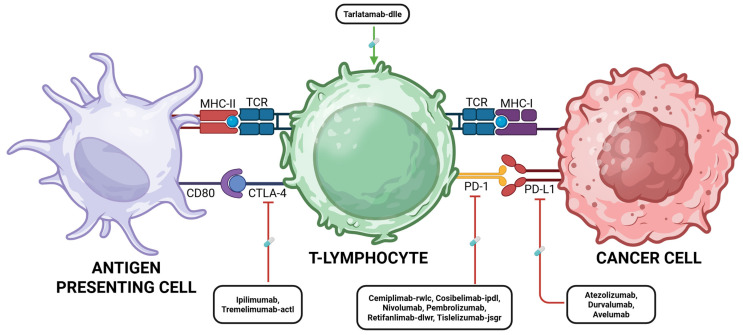
Immune checkpoint pathways in cancer. T-cell activation is modulated by inhibitory signals, including (Cytotoxic T-Lymphocyte-Associated Protein 4 (CTLA-4)—Cluster of Differentiation 80 (CD80) interactions between antigen-presenting cells and T lymphocytes, and PD-1–PD-L1 binding between T cells and tumor cells. Antibodies targeting CTLA-4 (ipilimumab, tremelimumab), PD-1 (nivolumab, pembrolizumab, cemiplimab, cosibelimab, retifanlimab, tislelizumab), and PD-L1 (atezolizumab, durvalumab, avelumab) restore antitumor immunity. Emerging bispecific agents such as tarlatamab are also shown. MHC—molecular histocompatibility complex; TCR—T-cell receptor; PD-1—Programmed cell death protein 1 (created with Biorender.com accessed on 20 September 2025).

Therapeutic disruption of PD-1/PD-L1 signaling through monoclonal antibodies has yielded unprecedented clinical benefit. Agents targeting PD-1 (pembrolizumab, nivolumab) and PD-L1 (atezolizumab, durvalumab, avelumab) restore T cell function and enhance antitumor immunity and have demonstrated durable responses and survival advantages across a broad spectrum of malignancies, including melanoma, non-small cell lung carcinoma (NSCLC), renal cell carcinoma, urothelial carcinoma, and triple-negative breast carcinoma [72,73]. Importantly, biomarkers such as PD-L1 expression levels, microsatellite instability (MSI) status, and tumor mutational burden (TMB) have emerged as predictive indicators of response and are increasingly incorporated into therapeutic decision-making [74]. A schematic overview of the PD-1/PD-L1 axis and the sites of action of clinically approved checkpoint inhibitors is depicted in Figure 3.

#### 3.3.2. CTLA-4 Pathway

Cytotoxic T-lymphocyte-associated protein 4 (CTLA-4) is an inhibitory receptor expressed on activated T cells and regulatory T cells, functioning primarily at the priming phase of T-cell activation within lymphoid organs. CTLA-4 competes with the costimulatory receptor CD28 for binding to B7 ligands (CD80/CD86) on antigen-presenting cells (APCs). By outcompeting CD28 and delivering inhibitory signals, CTLA-4 attenuates early T-cell activation, limits clonal expansion, and promotes peripheral tolerance—mechanisms co-opted by tumors to suppress antitumor immunity [75,76].

Blockade of CTLA-4 with the monoclonal antibody ipilimumab was the first immune checkpoint strategy to demonstrate an overall survival benefit in advanced melanoma, establishing a new therapeutic paradigm. Subsequent studies have shown that combined inhibition of CTLA-4 and PD-1 yields synergistic enhancement of T-cell effector function, leading to improved survival outcomes but also heightened immune-related toxicity [77]. Clinical applications of CTLA-4 blockade, alone or in rational combination regimens, are now being investigated across a wide range of tumor types. A schematic representation of the CTLA-4 signaling pathway, with therapeutic agents highlighted at their molecular targets, is presented in Figure 3.

## 4. FDA-Approved Targeted Therapies by Tumor Type

### 4.1. Tumor-Agnostic Therapies

Tumor-agnostic (tumor of origin-agnostic) therapies—also known as tissue-agnostic or site-agnostic treatments—are a groundbreaking class of cancer therapies approved based on specific molecular biomarkers rather than the anatomical origin of the tumor. This precision-medicine strategy allows treatments to be matched to genetic alterations such as mutations, fusions, or immune signatures, enabling highly personalized therapy across diverse cancer types.

The first FDA approval of this kind was pembrolizumab (Keytruda) in 2017, granted for any unresectable or metastatic solid tumor displaying microsatellite instability-high (MSI-H) or deficient mismatch repair (dMMR)—marking a pivotal shift from tumor-site to biomarker-driven oncology [78].

Since then, additional agents have secured tumor-agnostic FDA indications, including targeted therapies for NTRK gene fusions, BRAF V600E mutations, RET fusions, as well as treatments based on high tumor mutational burden (TMB-H) or HER2 overexpression (Table 2) [79,80].

### 4.2. Lung Cancer

Lung cancer remains the leading cause of cancer-related mortality worldwide, with non-small cell lung cancer (NSCLC) accounting for approximately 85% of cases and small cell lung cancer (SCLC) representing the remaining 15% [81]. The advent of targeted therapies and immunotherapies has dramatically transformed treatment paradigms in both groups, providing options that are more precise and effective compared with traditional chemotherapy. Molecular profiling is standard of care in NSCLC, as multiple oncogenic drivers and immune biomarkers guide the selection of targeted and immune checkpoint therapies. Targeted therapies in lung cancer can be broadly categorized into (1) kinase inhibitors directed against specific oncogenic drivers (EGFR, ALK, ROS1, RET, MET, BRAF, KRAS, HER2, NTRK); (2) anti-angiogenic therapies targeting VEGF/VEGFR; and (3) immune checkpoint inhibitors acting on PD-1, PD-L1, and CTLA-4 pathways [82,83,84,85]. These categories reflect a shift toward personalized treatment selection, informed by comprehensive molecular testing, which is now a standard component of lung cancer management (Table 3).

In NSCLC, one of the most important therapeutic breakthroughs has been the development of tyrosine kinase inhibitors (TKIs) directed against activating mutations in the *epidermal growth factor receptor (EGFR)* gene [82]. First-generation TKIs such as erlotinib and gefitinib demonstrated initial efficacy, followed by second-generation inhibitors like afatinib and dacomitinib, and ultimately third-generation osimertinib, which also overcomes the common resistance mutation T790M. Parallel advances have occurred in patients with gene rearrangements such as *ALK* and *ROS1*, where inhibitors including crizotinib, ceritinib, alectinib, brigatinib, lorlatinib, and entrectinib have achieved unprecedented clinical benefits. More recently, therapies targeting *RET* fusions (selpercatinib, pralsetinib) and *MET* exon 14 skipping mutations (capmatinib, tepotinib, savolitinib) have expanded the treatment arsenal [86,87,88].

Another clinically relevant category involves BRAF V600E mutations, for which combined inhibition with dabrafenib (BRAF inhibitor) and trametinib (MEK inhibitor) has become the standard of care [54]. Similarly, the recognition of *KRAS G12C* mutations as actionable targets has led to the approval of novel covalent inhibitors, such as sotorasib and adagrasib, offering new options for a historically undruggable oncogene [51,89]. Additional therapeutic directions include HER2-directed therapies in *ERBB2*-mutant NSCLC (trastuzumab deruxtecan, poziotinib in development) and NTRK inhibitors (entrectinib, larotrectinib) for patients with rare fusions [57]. Angiogenesis inhibition remains a key therapeutic pillar, particularly in advanced NSCLC. Bevacizumab, ramucirumab, and nintedanib act by blocking the vascular endothelial growth factor (VEGF) axis, limiting tumor vascularization and enhancing the efficacy of chemotherapy and immunotherapy backbones [84,90]. Equally transformative has been the rise of immune checkpoint inhibitors (ICIs), which have dramatically reshaped the treatment landscape for both NSCLC and SCLC. Monoclonal antibodies against PD-1 (nivolumab, pembrolizumab) and PD-L1 (atezolizumab, durvalumab, avelumab) have demonstrated durable responses and survival benefits, particularly in tumors with high PD-L1 expression, high tumor mutational burden (TMB), or microsatellite instability (MSI) [85]. In addition, the CTLA-4 inhibitor ipilimumab, alone or in combination with PD-1 blockade, has shown clinical activity, providing further immunotherapeutic strategies [91].

In contrast, treatment options for small cell lung cancer (SCLC) remain more limited, reflecting the aggressive biology and paucity of actionable driver mutations. Nevertheless, immunotherapy has emerged as a meaningful advance: the addition of PD-L1 inhibitors such as atezolizumab or durvalumab to first-line chemotherapy has improved outcomes and set a new standard of care. Emerging research exploring DLL3-targeted therapies (e.g., tarlatamab) has shown promising results in second-line treatment for ES-SCLC, with response rates up to 40% and a median overall survival of 14.3 months [92].

SCLC is characterized by rapid growth, early metastasis, and a high rate of initial response to chemotherapy, but relapse is common. Historically, treatment was limited to chemotherapy and radiotherapy [93]. In recent years, the addition of immunotherapy has improved outcomes for selected patients (Table 4).

### 4.3. Breast Cancer

Breast cancer is a heterogeneous group of malignant diseases that differ in histological, molecular, and clinical characteristics. Traditional classification is based on histology, with invasive ductal carcinoma being the most common type, while less frequent variants such as invasive lobular carcinoma and in situ lesions (ductal carcinoma in situ, lobular carcinoma in situ) are important for early diagnosis and monitoring [94]. However, molecular classification—based on the expression of hormone receptors (estrogen and progesterone), HER2 receptor status, and the proliferation index (Ki-67)—is the key factor guiding systemic therapy and predicting disease outcomes [95].

Based on these molecular features, breast cancer is commonly divided into four major subtypes: luminal A, luminal B, HER2-positive, and triple-negative breast cancer (TNBC). Luminal A tumors are characterized by strong hormone receptor expression with low proliferation, whereas luminal B tumors also express hormone receptors but have higher Ki-67 and/or HER2 positivity. The HER2-positive subtype is defined by HER2 overexpression or amplification in the absence of hormone receptors. Triple-negative cancers lack estrogen, progesterone, and HER2 receptors, and represent the most biologically aggressive group, prone to early metastasis and with limited therapeutic options [96,97].

Advances in molecular biology have enabled the identification of critical signaling pathways involved in breast cancer development and progression, leading to the introduction of targeted therapies. The most relevant therapeutic targets include the estrogen receptor pathway, the HER2 (ERBB2) pathway, cell cycle regulation via cyclins and cyclin-dependent kinases (CDKs), the PI3K/AKT/mTOR signaling axis, DNA repair mechanisms including BRCA mutations, as well as immunosuppressive pathways mediated by PD-1/PD-L1 interactions [6,56,65,66,67,68]. For patients with tumors that express the estrogen receptor, endocrine therapy remains the cornerstone of treatment. Selective estrogen receptor modulators (SERMs) such as tamoxifen and toremifene act as competitive antagonists of the receptor in breast tissue. Aromatase inhibitors, including anastrozole, letrozole, and exemestane, block peripheral estrogen synthesis, while selective estrogen receptor degraders (SERDs), such as fulvestrant and newer agents like elacestrant, directly induce receptor degradation [67,68]. In premenopausal patients, ovarian suppression can be achieved with LHRH agonists such as goserelin. HER2-positive breast cancers respond to monoclonal antibodies such as trastuzumab, pertuzumab, and newer derivatives like margetuximab [98]. To further enhance efficacy, antibody–drug conjugates (ADCs) such as ado-trastuzumab emtansine and trastuzumab deruxtecan have been developed and are now widely used in clinical practice. Additionally, oral HER2 tyrosine kinase inhibitors—including lapatinib, neratinib, and tucatinib—provide intracellular blockade of HER2 signaling [99]. CDK4/6 inhibitors (palbociclib, ribociclib, abemaciclib) have become the standard of care for HR+/HER2− metastatic breast cancer, in combination with endocrine therapy. These agents prevent cell cycle progression from the G1 to the S phase, thereby slowing tumor growth [100]. Dysregulation of the PI3K/AKT/mTOR signaling pathway, particularly through PIK3CA mutations, is common in HR+ subtypes and is associated with resistance to endocrine therapy. PI3K inhibitors (alpelisib, inavolisib), the AKT inhibitor capivasertib, and the mTOR inhibitor everolimus represent therapeutic strategies in this setting [101].

**Figure 4:**
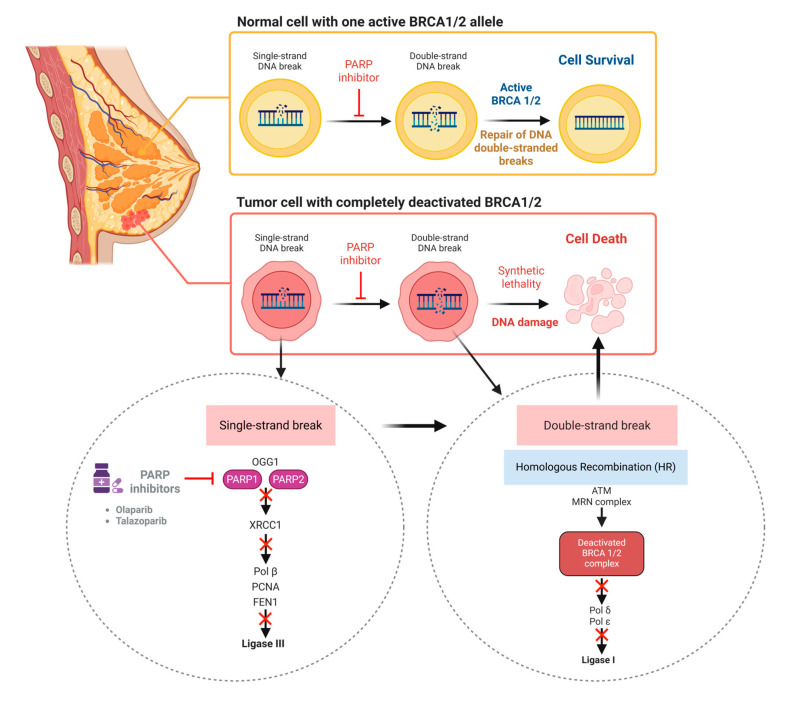
Therapeutic mechanism of PARP inhibitors. PARP inhibitors disable the single-strand break repair mechanism via **Ligase III**, in normal cells and tumor cells alike. This leads to progression to double-strand DNA breaks. In normal cells, the BRCA-mediated pathway activates and repairs the damage via homologous recombination, mediated by **Ligase I**. In tumor cells with inactivated BRCA, this mechanism cannot initiate the repair, which leads to further DNA degradation and inevitably apoptosis; PARP—poly (ADP-ribose) polymerase, HR—homologous recombination, PCNA—proliferating cell nuclear antigen, MRN—MRE11-RAD50-NBS1 complex (created with Biorender.com accessed on 20 September 2025).

For triple-negative breast cancer, particularly those with BRCA1/2 mutations or other homologous recombination deficiencies, poly (ADP-ribose) polymerase (PARP) inhibitors such as olaparib and talazoparib are effective (Figure 4) [102]. Although tumors with homologous recombination deficiency initially respond to PARP inhibitors, resistance commonly develops through BRCA reversion mutations, epigenetic reactivation of DNA repair, loss of PARP1 function, and upregulation of drug efflux transporters. Understanding these resistance pathways, including mechanisms such as replication fork stabilization and engagement of alternative DNA repair processes, provides a foundation for designing combination therapies that can overcome resistance and improve the long-term efficacy of PARP inhibition.

In addition, immunotherapy with anti-PD-1 antibodies (pembrolizumab) has shown clinical benefit in patients whose tumors express PD-L1 [103]. Newer strategies include antibody–drug conjugates targeting TROP2, such as sacituzumab govitecan and datopotamab deruxtecan, which are especially valuable for resistant forms of disease [104].

All currently FDA-approved targeted therapies for breast cancer, along with their therapeutic targets, are summarized in Table 5. These agents have significantly prolonged survival, improved quality of life, and advanced the concept of personalized oncology in clinical practice.

### 4.4. Colorectal Cancer

Colorectal cancer (CRC) is a biologically and clinically heterogeneous disease, with treatment increasingly guided by molecular profiling in addition to traditional staging. Molecular markers such as RAS (KRAS/NRAS) mutations, BRAF mutations, HER2 amplification, microsatellite instability (MSI) or mismatch repair (MMR) status, and tumor mutational burden (TMB) have become central to therapy selection and prognosis [105]. The introduction of targeted therapies has transformed the management of metastatic CRC, expanding treatment beyond conventional chemotherapy. Key therapeutic strategies include targeting angiogenesis, inhibiting the EGFR pathway, blocking oncogenic drivers such as KRAS and BRAF, and harnessing immune checkpoint inhibition in biomarker-defined subgroups (Table 6).

From a molecular perspective, CRC development and progression are driven by alterations in several major signaling pathways. The MAPK/ERK pathway, frequently activated by KRAS and BRAF mutations, promotes uncontrolled proliferation and survival, and is therapeutically targeted with EGFR inhibitors (cetuximab, panitumumab) in RAS wild-type tumors and with BRAF inhibitors (encorafenib in combination with cetuximab) in BRAF V600E–mutant disease [4,106,107]. The PI3K/AKT/mTOR pathway, often altered through PIK3CA mutations or PTEN loss, regulates tumor growth and contributes to resistance against upstream inhibitors. WNT/β-catenin signaling, typically disrupted by APC mutations, is a hallmark of colorectal carcinogenesis, driving stemness and epithelial–mesenchymal transition, although direct pharmacologic targeting remains challenging [48,61]. In addition, angiogenesis signaling via VEGF/VEGFR plays a critical role in metastatic spread and is effectively targeted with agents such as bevacizumab, ramucirumab, ziv-aflibercept, and fruquintinib [108]. Finally, immune pathways, particularly MMR deficiency and MSI-high status, identify subgroups highly responsive to immune checkpoint inhibitors such as pembrolizumab and nivolumab, with ipilimumab as a complementary CTLA-4 blockade [109,110].

### 4.5. Prostate Cancer

Prostate cancer is the most common malignancy among men in the United States and a leading cause of cancer-related death [111]. Based on the latest research, the molecular mechanisms of prostate cancer (PCa) are complex and involve a variety of genetic, epigenetic, and signaling pathway alterations. Androgen Receptor (AR) Signaling is crucial in both the initiation and progression of PCa. Androgens bind to ARs, promoting transcription of genes that support cell survival and proliferation. However, in certain cases, despite androgen deprivation therapy (ADT), cancer could progress to Castration-Resistant Prostate Cancer (CRPC) due to: AR gene amplification, AR mutations and splice variants, intratumoral androgen synthesis or activation of AR by other signaling pathways. Genomic alterations play a crucial role in the development of PCa. There are common mutations in SPOP, FOXA1, TP53, PTEN, RB1, MYC, and other oncogene genes, while mutations linked to DNA repair defects are present at BRCA1/2 and ATM genes. On the other hand, Copy Number Alterations (CNAs) affect AR, MYC, and other oncogenes. Key signaling pathways involved in the development of PCa include PI3K/AKT/mTOR Pathway, Wnt/β-catenin Pathway, MAPK Pathways, and STAT3 and EZH2 Pathways, as well as alterations in cell cycle regulators, such as in CDK12 and RB1, that contribute to unchecked proliferation. Management has evolved significantly over the past decade, with the development of targeted therapies and next-generation hormonal agents that address key molecular drivers of disease progression (Table 7). While androgen deprivation therapy (ADT) remains the cornerstone of treatment, new agents targeting the androgen receptor pathway, DNA repair defects, bone metastases, and other molecular vulnerabilities have expanded therapeutic options.

The development of targeted therapies for prostate cancer reflects the central role of the androgen receptor (AR) signaling pathway, along with the recognition of molecular vulnerabilities such as DNA repair deficiencies and prostate-specific antigens. One of the key advances has been the introduction of next-generation hormonal agents that directly or indirectly inhibit AR activity. Abiraterone acetate blocks androgen biosynthesis by inhibiting CYP17, while apalutamide, enzalutamide, and darolutamide act as potent AR antagonists, effectively suppressing tumor growth even in the castration-resistant state, particularly in metastatic castration-sensitive prostate cancer (mCSPC) [112]. A comparative analysis of two androgen receptor inhibitors—enzalutamide (Xtandi) and darolutamide (Nubeqa)—showed that enzalutamide significantly delayed radiographic progression-free survival (rPFS) by nearly 50% compared to darolutamide in patients with metastatic hormone-sensitive prostate cancer (mHSPC) [113]. This was based on a matching-adjusted indirect comparison (MAIC) of the ARCHES and ARANOTE trials.

Androgen deprivation therapy (ADT) remains a cornerstone of prostate cancer management, traditionally achieved through surgical castration or more commonly with LHRH agonists such as leuprolide, goserelin, and triptorelin [114]. The GnRH antagonist degarelix provides a rapid and reversible method of testosterone suppression, while the oral agent relugolix offers a convenient alternative with similar efficacy. These approaches are essential in both localized high-risk disease and advanced metastatic settings, forming the foundation upon which newer therapies are layered [115].

For patients with homologous recombination repair (HRR) gene mutations, PARP inhibitors have introduced a new level of precision. Olaparib, rucaparib, talazoparib, and the dual formulation of niraparib with abiraterone are approved options for men with BRCA1/2 or related DNA repair defects. These agents exploit synthetic lethality, selectively targeting cancer cells while sparing normal tissue [116]. Finally, in patients who progress after androgen receptor-directed therapy and docetaxel, cabazitaxel, a next-generation taxane chemotherapy, provides meaningful benefit. Although not molecularly targeted in the classical sense, cabazitaxel is included among advanced prostate cancer treatments because of its specific activity against resistant disease [117]. Theranostics combines diagnostics and therapy using radiopharmaceuticals that target prostate-specific membrane antigen (PSMA). The most notable agent is Pluvicto, which uses gallium-68 for imaging and lutetium-177 for treatment. Newer trials are exploring actinium-225, which emits more potent alpha particles for deeper tumor penetration [118].

Kairos Pharma’s ENV105 is a resistance-reversing agent targeting CD105, a protein linked to treatment resistance. It is in Phase 2 trials for metastatic castration-resistant prostate cancer and shows promise in restoring the effectiveness of existing therapies like enzalutamide [119].

### 4.6. Gastric Cancer

Gastric cancer remains a major global health challenge, often diagnosed at an advanced stage when curative surgery is not possible [120]. Traditional chemotherapy regimens have long been the backbone of systemic treatment, but advances in molecular oncology and immunotherapy have expanded the therapeutic armamentarium (Table 8). Today, targeted agents directed against HER2, angiogenesis pathways, Claudin 18.2, and immune checkpoints play a central role in the management of advanced gastric and gastroesophageal junction cancers [121].

HER2 overexpression, present in a subset of gastric cancers, has provided one of the earliest and most impactful targets. Trastuzumab, a monoclonal antibody against HER2, is approved in combination with chemotherapy as the first targeted therapy to improve survival in HER2-positive gastric cancer. Building on this success, fam-trastuzumab deruxtecan, an antibody–drug conjugate, has demonstrated remarkable activity in patients who progress after trastuzumab-based therapy, offering a new line of effective treatment. Angiogenesis inhibition also has a defined role in gastric cancer. Ramucirumab, a monoclonal antibody targeting VEGFR2, is approved both as monotherapy and in combination with paclitaxel for patients with previously treated advanced disease [98,122]. Its approval was a major advance in the second-line setting, improving survival in a population with limited options.

In recent years, immunotherapy has reshaped gastric cancer treatment. PD-1 inhibitors, including nivolumab, pembrolizumab, and, more recently tislelizumab, are approved in biomarker-selected populations. Nivolumab has shown benefit in combination with chemotherapy in HER2-negative advanced gastric cancer, while pembrolizumab is used in PD-L1-positive tumors and microsatellite instability-high (MSI-H) cancers [123]. Tislelizumab, a PD-1 inhibitor recently approved, further expands the options available for immune checkpoint blockade in this disease. Another important breakthrough is the development of zolbetuximab, the first-in-class antibody targeting Claudin 18.2, a tight junction protein expressed in a subset of gastric and gastroesophageal junction cancers. Approved in combination with chemotherapy, zolbetuximab offers a novel biomarker-driven therapy for patients with CLDN18.2-positive tumors, marking a new step toward personalized treatment in gastric cancer [124]. Durvalumab (Imfinzi), an anti-PD-L1 monoclonal antibody, has shown significant promise in treating resectable gastric and gastroesophageal junction (GEJ) cancers, based on the results of the MATTERHORN Phase III trial presented at ASCO 2025. MATTERHORN Phase III trial included 948 patients with stage II–IVA resectable gastric or GEJ adenocarcinoma who were randomized to receive either: Durvalumab + FLOT chemotherapy (perioperative: 2 neoadjuvant + 2 adjuvant cycles, followed by 10 cycles of durvalumab monotherapy), Placebo + FLOT, followed by placebo maintenance. Event-Free Survival (EFS) 24-month EFS: 67.4% (durvalumab) vs. 58.5% (placebo), while Hazard Ratio: 0.71 (95% CI: 0.58–0.86), *p* < 0.001 [125]. FDA Priority Review & Breakthrough Therapy Designation was granted for Durvalumab in July 2025, and approval decision is expected in Q4 2025. Durvalumab combined with FLOT chemotherapy is poised to redefine the standard of care for resectable gastric and GEJ cancers. It is the first immunotherapy regimen to show statistically significant improvements in EFS and Pathologic Complete Response (pCR) in this setting [126].

### 4.7. Liver and Bile Duct Cancers

Primary liver cancers, most commonly hepatocellular carcinoma (HCC), and bile duct cancers (cholangiocarcinomas) are aggressive malignancies often diagnosed at advanced stages [127]. For many years, systemic treatment options were limited, with sorafenib as the only approved therapy. However, the past decade has seen remarkable progress with the introduction of targeted therapies and immunotherapies that have redefined the standard of care (Table 9).

In hepatocellular carcinoma, angiogenesis plays a critical role in tumor biology, and VEGF pathway inhibitors have been central to treatment. Sorafenib, the first agent to demonstrate survival benefit, remains an option, while lenvatinib, regorafenib, and cabozantinib offer alternatives or second-line therapy. Bevacizumab, an anti-VEGF antibody, in combination with the PD-L1 inhibitor atezolizumab, has become a frontline standard, providing superior survival compared with sorafenib. Ramucirumab, targeting VEGFR2, is another important therapy in biomarker-selected patients with elevated alpha-fetoprotein (AFP) [128].

Checkpoint inhibitors have transformed liver cancer management, particularly in immunotherapy-based combinations. Nivolumab and pembrolizumab are PD-1 inhibitors with established roles in HCC, while durvalumab, another PD-L1 inhibitor, is approved in combination with the CTLA-4 inhibitor tremelimumab. This dual checkpoint strategy enhances antitumor immunity and has provided durable responses for a subset of patients. Ipilimumab and tremelimumab, in combination with PD-1/PD-L1 agents, further expand the immunotherapy landscape in advanced liver cancer [129].

For bile duct cancers, precision oncology has played a transformative role. FGFR2 fusions are actionable targets in intrahepatic cholangiocarcinoma, with pemigatinib and futibatinib now approved in this setting. IDH1 mutations, present in a subset of cholangiocarcinomas, can be treated with ivosidenib, providing an additional biomarker-driven approach. More recently, zanidatamab, a HER2-targeted bispecific antibody, has been approved for HER2-amplified biliary tract cancers, addressing another clinically relevant molecular subset [130].

### 4.8. Thyroid Cancer

Thyroid cancer encompasses a heterogeneous group of malignancies, including differentiated thyroid cancers (papillary, follicular, and Hurthle cell), medullary thyroid cancer, and the aggressive anaplastic thyroid carcinoma. While surgery and radioactive iodine remain the cornerstones of therapy for most differentiated thyroid cancers, a subset of patients develop advanced or refractory disease that no longer responds to traditional approaches [131]. In these cases, targeted therapies have become essential, providing meaningful clinical benefit through inhibition of specific molecular pathways (Table 10).

The vascular endothelial growth factor (VEGF) signaling pathway is a central driver of thyroid tumor growth and angiogenesis. Multikinase inhibitors such as sorafenib and lenvatinib have become standard options for radioactive iodine–refractory differentiated thyroid cancer, significantly prolonging progression-free survival. Cabozantinib, another multikinase inhibitor with activity against VEGFR, MET, and RET, is approved for medullary thyroid cancer and as a second-line option in differentiated thyroid cancer. Vandetanib similarly targets VEGFR, EGFR, and RET and is approved for advanced medullary thyroid cancer, providing durable disease control in this rare subtype [132,133].

The discovery of actionable genetic alterations has further refined thyroid cancer therapy. RET fusions and mutations, common in medullary and some papillary thyroid cancers, can be effectively treated with selective RET inhibitors such as selpercatinib and pralsetinib, which have demonstrated high response rates and favorable tolerability compared to older multikinase inhibitors. For patients with anaplastic thyroid carcinoma harboring the BRAF V600E mutation, the combination of dabrafenib (a BRAF inhibitor) and trametinib (a MEK inhibitor) provides an important targeted option, dramatically improving outcomes in a disease that previously had a dismal prognosis [134,135].

### 4.9. Bladder Cancer

Bladder cancer, most often presenting as urothelial carcinoma, is a complex malignancy historically treated with surgery, platinum-based chemotherapy, and intravesical therapy for early-stage disease [126]. In recent years, advances in molecular biology and immuno-oncology have led to the approval of several targeted therapies and immunotherapies (Table 11), particularly for patients with advanced or metastatic disease who progress after standard chemotherapy. These treatments have substantially expanded the therapeutic landscape, providing durable responses and improving survival in selected patient populations [136].

A breakthrough has been the introduction of immune checkpoint inhibitors, which harness the body’s own immune system to fight cancer. Agents such as atezolizumab, avelumab, nivolumab, and pembrolizumab target the PD-1/PD-L1 pathway, restoring T-cell activity against tumor cells. These therapies are approved for advanced urothelial carcinoma in patients who have failed prior chemotherapy, and in some cases as first-line treatment for cisplatin-ineligible patients with PD-L1-positive tumors [137]. Avelumab also has a unique role as maintenance therapy after first-line platinum-based chemotherapy, prolonging overall survival compared with best supportive care.

In addition to checkpoint blockade, antibody–drug conjugates have become an important treatment option. Enfortumab vedotin, directed against Nectin-4 and linked to a cytotoxic agent, has demonstrated meaningful activity in patients previously treated with chemotherapy and immunotherapy. Its approval has been a major step forward for those with limited remaining treatment options. Targeted small-molecule inhibitors also contribute to precision therapy in bladder cancer. Erdafitinib, an oral fibroblast growth factor receptor (FGFR) inhibitor, is approved for tumors harboring susceptible FGFR2 or FGFR3 alterations. By blocking aberrant FGFR signaling, erdafitinib provides benefit in a biomarker-defined population that historically had poor responses to standard therapy [138]. More recently, immunotherapy innovation has expanded further with nogapendekin alfa inbakicept, an IL-15 superagonist designed to enhance natural killer and T-cell activity [139]. Its approval for BCG-unresponsive non-muscle invasive bladder cancer represents an important alternative for patients who might otherwise require radical surgery.

### 4.10. Renal Cell Carcinoma

Renal cell carcinoma (RCC), has historically been resistant to conventional chemotherapy and radiation [140]. Over the past two decades, a deeper understanding of tumor biology—particularly angiogenesis, hypoxia-inducible pathways, and immune evasion—has led to the development of multiple targeted therapies and immunotherapies that have transformed patient outcomes (Table 12). These agents, used either as monotherapy or in combination, form the backbone of treatment for advanced and metastatic RCC [140].

One of the earliest therapeutic breakthroughs was the inhibition of angiogenesis, a hallmark of RCC. Agents such as bevacizumab, an anti-VEGF antibody, and multiple VEGF receptor tyrosine kinase inhibitors (TKIs), including sunitinib, sorafenib, pazopanib, axitinib, cabozantinib, lenvatinib, and tivozanib, have demonstrated robust clinical activity. These drugs block the VEGF signaling cascade, reducing blood supply to tumors and slowing progression. Their development marked a turning point in kidney cancer therapy, shifting the standard of care away from nonspecific immunotherapies such as interferon. Beyond VEGF inhibition, additional pathways have been successfully targeted [140,141]. The hypoxia-inducible factor 2α (HIF-2α) inhibitor belzutifan provides a novel option for patients with von Hippel–Lindau disease-associated RCC and has expanded into broader RCC indications [142]. Immunotherapy has also revolutionized kidney cancer management. The checkpoint inhibitors nivolumab and pembrolizumab, targeting PD-1, have shown durable responses, while ipilimumab, a CTLA-4 inhibitor, is used in combination with PD-1 blockade to enhance T-cell activity. Avelumab, a PD-L1 inhibitor, also plays a role in combination regimens with TKIs such as axitinib [140,143]. These immune checkpoint inhibitors have redefined the therapeutic landscape, providing long-term disease control for a subset of patients previously considered incurable.

### 4.11. Leukemias

Leukemia is a heterogeneous group of hematologic malignancies arising from the bone marrow and blood. Treatment strategies vary by disease subtype—acute myeloid leukemia (AML), acute lymphoblastic leukemia (ALL), chronic myeloid leukemia (CML), and chronic lymphocytic leukemia (CLL)—and are increasingly guided by molecular biomarkers and immunophenotyping [144]. The introduction of targeted therapies, including tyrosine kinase inhibitors (TKIs), monoclonal antibodies, antibody–drug conjugates, small-molecule inhibitors, and cellular immunotherapies, has transformed outcomes across leukemias (Table 13). The introduction of targeted therapies has revolutionized the treatment of leukemias, shifting the focus from conventional cytotoxic chemotherapy to precision medicine tailored to molecular drivers and immune characteristics. In chronic myeloid leukemia (CML), the discovery of the BCR-ABL fusion gene established tyrosine kinase inhibitors as the standard of care. Imatinib was the first to transform outcomes, and it has since been followed by more potent agents such as dasatinib, nilotinib, bosutinib, ponatinib, and the allosteric inhibitor asciminib, which address resistance mutations, including T315I [145]. In acute myeloid leukemia (AML), a different spectrum of targeted therapies has emerged, particularly for patients with FLT3 mutations [146]. Agents such as midostaurin, gilteritinib, and quizartinib inhibit aberrant FLT3 signaling, while enasidenib, ivosidenib, and olutasidenib target IDH2 and IDH1 mutations, restoring normal differentiation pathways. The menin inhibitor revumenib represents an important advance for KMT2A-rearranged and NPM1-mutated leukemias, further broadening the therapeutic landscape [147,148].

Differentiation therapy remains a cornerstone for specific AML subsets, most notably acute promyelocytic leukemia, where tretinoin (all-trans retinoic acid) induces maturation of malignant promyelocytes [149]. Additional agents such as glasdegib, which targets the Hedgehog signaling pathway, further reflect the trend toward pathway-directed strategies in AML [150]. In parallel, therapies directed at B-cell surface antigens have dramatically improved the outlook for lymphoid leukemias. Anti-CD20 antibodies, including rituximab, obinutuzumab, and ofatumumab, are central in chronic lymphocytic leukemia (CLL), while alemtuzumab, which targets CD52, remains an option for refractory disease [151].

Cellular immunotherapy has opened an entirely new chapter in the treatment of leukemias. Chimeric antigen receptor (CAR) T-cell therapies such as tisagenlecleucel, brexucabtagene autoleucel, lisocabtagene maraleucel, and obecabtagene autoleucel harness the patient’s own immune system to recognize and eradicate CD19-expressing blasts in relapsed or refractory ALL and certain lymphoid leukemias. Complementing these, tagraxofusp, a CD123-directed fusion protein, addresses rare leukemic presentations such as blastic plasmacytoid dendritic cell neoplasm [152,153].

### 4.12. Pancreatic Cancer

Pancreatic cancer, particularly pancreatic ductal adenocarcinoma (PDAC), remains one of the most lethal malignancies, with limited curative options and a poor overall prognosis [154]. While cytotoxic chemotherapy continues to serve as the cornerstone of treatment, advances in molecular profiling have revealed actionable alterations in a subset of patients, enabling the use of targeted therapies (Table 14). These developments represent an important step toward individualized care in a disease long resistant to therapeutic advances.

One of the earliest targeted agents approved for pancreatic cancer is erlotinib, an EGFR tyrosine kinase inhibitor, used in combination with gemcitabine. Although the survival benefit is modest, it marked the beginning of targeted approaches in this cancer type [155]. For patients with germline BRCA1/2 mutations, the PARP inhibitor olaparib has changed clinical practice. Approved as maintenance therapy for metastatic disease following platinum-based chemotherapy, olaparib leverages synthetic lethality to exploit DNA repair deficiencies, extending survival in biomarker-selected patients [156]. Everolimus, an mTOR inhibitor, and sunitinib, a multikinase inhibitor, are primarily approved for pancreatic neuroendocrine tumors (pNETs) rather than pancreatic adenocarcinoma. Both agents significantly improve progression-free survival and remain key options in the management of advanced pNETs, highlighting the importance of distinguishing tumor histologies within the pancreas [157]. More recent advances include belzutifan, a HIF-2α inhibitor approved for von Hippel–Lindau (VHL)-associated pancreatic neuroendocrine tumors, further expanding the precision oncology toolbox in hereditary cancer syndromes [158]. Additionally, zenocutuzumab, a bispecific antibody targeting HER2 and HER3, is approved for tumors with NRG1 fusions, a rare but actionable alteration occasionally found in pancreatic cancer [159].

Although the proportion of patients eligible for these therapies remains relatively small, the approval of EGFR, PARP, VEGF/mTOR pathway inhibitors, and NRG1-targeted therapies demonstrates a growing ability to personalize treatment. Ongoing research into KRAS inhibitors, immunotherapy combinations, and other novel approaches promises to further broaden the scope of targeted therapy in pancreatic cancer.

### 4.13. Brain Tumors

Brain cancer represents a heterogeneous group of malignant diseases that differ in histological, molecular, and clinical features. While traditional classification relied primarily on histology—such as glioblastoma (GMB), oligodendroglioma, and astrocytoma—modern diagnostic standards now place molecular characteristics at the center of glioma classification [34]. In fact, the current WHO classification of central nervous system tumors mandates integration of molecular markers, including IDH1/2 mutations, 1p/19q codeletion status, ATRX and TP53 alterations, and MGMT promoter methylation, as essential criteria for accurate diagnosis. In some instances, such as differentiating oligodendroglioma grade 3 from glioblastoma, molecular testing (e.g., fluorescence in situ hybridization, FISH, for 1p/19q codeletion) is indispensable, as histology alone is insufficient to distinguish between entities with profoundly different prognosis and treatment strategies [34,160]. Advances in molecular biology have further enabled the identification of critical signaling pathways involved in tumorigenesis and progression of brain cancers, leading to the development of targeted therapies. Among the most important therapeutic targets are hypoxia-inducible factor (HIF) pathways, angiogenesis mediated by vascular endothelial growth factor (VEGF), MAPK signaling, the mTOR pathway, and mutant metabolic enzymes such as IDH1/2 [34,161,162,163].

Several targeted agents have been approved for the treatment of brain tumors, particularly in recurrent or molecularly defined settings (Table 15). These agents have significantly expanded the therapeutic landscape by allowing for more personalized treatment approaches. While traditional chemotherapy and radiotherapy remain the backbone of therapy, targeted treatments provide meaningful benefits in tumor control, progression-free survival, and quality of life, particularly in biomarker-driven subgroups.

EGFR inhibitors (e.g., erlotinib, afatinib) and EGFRvIII-targeted vaccines have shown limited success in glioblastoma patients, due to poor BBB penetration and intratumoral heterogeneity. At the same time, Bevacizumab, an anti-VEGF monoclonal antibody, is FDA-approved for recurrent GBM. It improves progression-free survival but not overall survival. On the other hand, Vorasidenib, an oral Isocitrate Dehydrogenase (IDH1/2) inhibitor, recently approved for IDH-mutant gliomas, significantly prolongs progression-free survival [164].

### 4.14. Skin Cancers

Skin cancers represent a diverse group of malignancies that include melanoma, cutaneous squamous cell carcinoma, basal cell carcinoma, and rare entities such as uveal melanoma and cutaneous T-cell lymphoma [165]. While surgery and radiotherapy remain central for localized disease, the management of advanced and metastatic skin cancers has been revolutionized by targeted therapies and immunotherapies (Table 16). These agents exploit specific oncogenic drivers or modulate immune checkpoint pathways, leading to durable responses in patients with historically limited options.

In melanoma, the discovery of activating mutations in the BRAF gene (V600E/K) has transformed treatment. BRAF inhibitors such as vemurafenib, dabrafenib, and encorafenib, particularly when combined with MEK inhibitors like trametinib, cobimetinib, or binimetinib, provide robust and durable responses while delaying resistance [166]. For patients without BRAF mutations, immune checkpoint inhibitors, including anti-PD-1 antibodies (nivolumab, pembrolizumab, cemiplimab, cosibelimab, retifanlimab) and CTLA-4 inhibition (ipilimumab), have redefined outcomes, either as monotherapy or in combinations such as nivolumab–relatlimab (targeting PD-1 and LAG-3) [143]. Cutaneous squamous cell carcinoma (cSCC) has shown remarkable sensitivity to PD-1 inhibitors, with cemiplimab and pembrolizumab approved as standard therapies [167]. These agents highlight the profound role of immunotherapy in non-melanoma skin cancers. Basal cell carcinoma (BCC), driven in many cases by aberrant Hedgehog pathway signaling, is effectively treated with smoothened (SMO) inhibitors such as vismodegib and sonidegib [168]. These drugs directly target the molecular drivers of tumor growth, representing a precision approach in advanced BCC resistant to local therapy.

For rare skin malignancies, specialized agents have emerged. Alitretinoin, a retinoid, is used for cutaneous T-cell lymphoma [169]. Tebentafusp, a bispecific fusion protein targeting gp100 and CD3, is approved for uveal melanoma, marking the first therapy to demonstrate an overall survival benefit in this rare disease [170]. Additionally, avelumab and atezolizumab have roles in Merkel cell carcinoma, a highly aggressive but immunotherapy-sensitive skin cancer [171].

### 4.15. Ovarian Epithelial, Fallopian Tube, and Primary Peritoneal Cancers

Ovarian epithelial cancers, along with closely related fallopian tube and primary peritoneal cancers, represent the majority of gynecologic malignancies in this category [172]. These cancers are often diagnosed at an advanced stage, and while surgery and platinum-based chemotherapy remain central components of treatment, the development of targeted therapies has significantly changed the therapeutic landscape (Table 17). Precision medicine approaches now allow clinicians to tailor therapy based on molecular alterations and tumor biology, improving both survival and quality of life.

One of the most impactful advances has been the introduction of PARP inhibitors for tumors with homologous recombination deficiency (HRD), including BRCA1/2 mutations. Olaparib, niraparib, and rucaparib are approved for maintenance therapy following chemotherapy response, as well as for recurrent disease in biomarker-selected populations. These agents exploit synthetic lethality, blocking DNA repair pathways and leading to selective tumor cell death in HRD-positive cancers. Their integration into frontline and recurrent settings has transformed the long-term management of ovarian cancer [173]. Another important therapeutic class is angiogenesis inhibition. Bevacizumab, a VEGF-targeting monoclonal antibody, is approved both as maintenance therapy in the frontline setting and as part of treatment for recurrent disease [141]. By disrupting tumor vascular supply, bevacizumab slows disease progression and provides additional benefit when combined with chemotherapy.

A more recent innovation is the use of antibody–drug conjugates (ADCs). Mirvetuximab soravtansine, an ADC targeting folate receptor alpha (FRα), was recently approved for platinum-resistant ovarian cancer with high FRα expression [174]. This agent delivers a cytotoxic payload directly into tumor cells, combining targeted precision with potent antitumor activity, and provides an important new option for patients with limited therapeutic alternatives.

### 4.16. Cancer of Unknown Primary (CUP)

A cancer of unknown primary (CUP) is defined as a biopsy-confirmed malignancy in which the primary site remains unidentified despite comprehensive pathological assessment and radiographic studies [175]. CUP accounts for roughly 5% of all cancers and carries a poor prognosis, with only about 30% of patients alive at one year. Current recommendations emphasize early molecular profiling, either from metastatic tissue or liquid biopsy, as a means of guiding treatment. In addition, artificial intelligence-based platforms, such as OncoOrigin, have shown potential in integrating molecular signatures to improve the detection of the tissue of origin and support more tailored therapeutic approaches [48].

## 5. Future Outlooks in Precision Oncology

The future of precision oncology undoubtedly lies in a multidimensional understanding of cancer biology that transcends traditional sequencing approaches. Emerging technologies such as spatial transcriptomics, liquid biopsy, and multi-omics are redefining how we analyze, monitor, and treat malignancies, bridging the gap between molecular data and clinical decision-making.

Spatial transcriptomics represents a paradigm shift in the study of tumor biology. Unlike conventional RNA-sequencing, which loses the spatial context of gene expression, this technique allows scientists to map transcriptional activity directly within the architecture of the tissue. By linking gene expression to precise cellular locations, spatial transcriptomics sheds light on the complex interactions between cancer cells and immune cells. This insight is particularly critical for understanding immune evasion, intratumoral heterogeneity, and drug resistance mechanisms. In the future, spatial single-cell analyses are expected to inform rational design of immunotherapies and identify microenvironment-specific therapeutic vulnerabilities [176].

Liquid biopsy has emerged as a minimally invasive yet highly informative complement to tissue-based genomic profiling. Through the analysis of circulating tumor DNA (ctDNA), liquid biopsy enables dynamic tracking of tumor evolution and therapeutic response. This approach offers unique advantages for patients where tissue acquisition is limited or unsafe, and it even holds promise for early cancer detection, minimal residual disease monitoring, and timely identification of resistance mutations. Advances in sensitivity, standardization, and bioinformatics pipelines will be essential for the broad clinical implementation of liquid biopsy as a real-time molecular window into tumor biology [177,178].

Multi-omics integration represents the ultimate step toward comprehensive cancer characterization. By combining genomic, transcriptomic, proteomic, metabolomic, and epigenomic layers, researchers can capture the complexity of oncogenic signaling pathways and identify synergistic biomarkers that individual omics analyses may overlook [179]. Integrative platforms powered by artificial intelligence and machine learning are becoming increasingly capable of identifying clinically meaningful patterns from these high-dimensional datasets, predicting therapeutic response, and stratifying patients based on molecular and phenotypic features. This comprehensive approach will enable a deeper understanding of tumor heterogeneity, clonal evolution, and the molecular basis of resistance to therapy [180].

In the coming years, the convergence of these technologies, supported by advanced computational frameworks, cloud-based data sharing, and harmonized biobanking, will accelerate the translation of discovery into practice. The integration of spatial, liquid biopsy, and multi-omics data will lead to highly advanced models capable of predicting disease trajectories and optimizing individualized therapy regimens [181]. Moreover, as technological costs decrease and regulatory frameworks mature, these methods will become accessible to a broader patient population, fostering equitable precision oncology worldwide.

## 6. Conclusions

Molecular profiling through whole-genome, whole-exome, and transcriptome sequencing has fundamentally transformed cancer diagnostics and treatment. By moving beyond traditional histopathological classification, these technologies enable precise identification of actionable alterations, discovery of novel biomarkers, and recognition of resistance mechanisms that shape therapeutic decision-making. The integration of genomic and transcriptomic data provides a more comprehensive view of tumor biology, supporting the development of highly individualized treatment strategies.

The rapidly expanding repertoire of FDA-approved targeted therapies underscores the clinical impact of precision oncology, allowing for genotype-directed interventions across a wide range of tumor types. These advances not only extend survival but also improve quality of life by matching patients to therapies most likely to be effective. Looking ahead, the future of precision cancer care lies in the integration of multi-omics approaches—including proteomics, metabolomics, and epigenomics—with advanced bioinformatics and artificial intelligence. Such innovations will refine the interpretation of complex datasets, accelerate biomarker discovery, and further personalize therapy. Equally important are efforts to broaden access to molecular testing and targeted treatments, ensuring that the benefits of precision oncology are equitably distributed.

The convergence of advanced sequencing technologies, expanding therapeutic options, and data-driven interpretation heralds a new era of oncology in which treatment is tailored to the unique molecular landscape of each patient’s tumor. In this context, artificial intelligence plays a pivotal role by enabling the integration of complex molecular and clinical datasets, improving the accuracy of tumor classification, and facilitating the prediction of therapeutic response. Together, these advances support a more precise and individualized approach to cancer care.

## Figures and Tables

**Table 1:** Common oncogenic driver genes, function, pathways affected, and associated cancer types.

Gene	Function	Pathway Affected	Associated Cancer Type(s)
***AKT1***	proto-oncogene	PI3K–AKT–mTOR pathway	breast, endometrial
***ALK***	proto-oncogene	RTK signaling	NSCLC, ALCL, neuroblastoma
***APC***	tumor suppressor	WNT/β-catenin	colorectal
***BRAF***	proto-oncogene	MAPK pathway	melanoma, colorectal, papillary thyroid
***CDKN2A***	tumor suppressor	CDK4/6–RB pathway	melanoma, pancreatic, glioblastoma
***CTNNB1***	proto-oncogene	WNT/β-catenin	HCC, endometrial, desmoid
***EGFR***	proto-oncogene	RTK; RAS–MAPK; PI3K–AKT pathway	NSCLC, glioblastoma
***ERBB2/HER2***	proto-oncogene	RTK; MAPK; PI3K–AKT pathway	breast, gastric cancer
***FGFR2/FGFR3***	proto-oncogene	RTK signaling	cholangiocarcinoma (FGFR2), bladder (FGFR3)
***KIT***	proto-oncogene	RTK signaling	GIST, melanoma (acral/mucosal)
***KRAS***	proto-oncogene	RAS–RAF–MEK–ERK (MAPK) pathway	lung adenocarcinoma, colorectal, pancreatic
***MET***	proto-oncogene	RTK → MAPK; PI3K–AKT pathway	NSCLC, papillary RCC
***NRAS***	proto-oncogene	MAPK pathway	melanoma, AML
***PDGFRA***	proto-oncogene	RTK signaling	GIST
***PIK3CA***	proto-oncogene	PI3K–AKT–mTOR pathway	breast, endometrial, HNSCC
***PTEN***	tumor suppressor	PI3K–AKT–mTOR pathway	prostate, endometrial, glioblastoma
***RB1***	tumor suppressor	Cell-cycle (G1/S checkpoint)	retinoblastoma, SCLC
***RET***	proto-oncogene	RTK signaling	medullary & papillary thyroid cancer, NSCLC
***ROS1***	proto-oncogene	RTK signaling	NSCLC
***SMAD4***	tumor suppressor	TGF-β signaling	pancreatic, colorectal
***TP53***	tumor suppressor	DNA damage response, cell cycle, apoptosis	sarcomas, brain tumors, adrenal gland cancer, and breast cancer
***VHL***	tumor suppressor	HIF pathway	clear-cell RCC

NSCLC—non-small cell lung cancer; SCLC—small-cell lung cancer; HNSCC—head and neck squamous cell carcinoma; HCC—hepatocellular carcinoma; RCC—renal cell carcinoma; GIST—gastrointestinal stromal tumor; AML—acute myeloid leukemia; ALCL—anaplastic large cell lymphoma; RTK—receptor tyrosine kinase; MAPK—mitogen-activated protein kinase; PI3K—phosphatidylinositol 3-kinase; AKT—protein kinase B; mTOR—mechanistic target of rapamycin; WNT—Wnt signaling pathway (Wingless/Int-1); TGF-β—transforming growth factor beta; HIF—hypoxia-inducible factor; CDK—cyclin-dependent kinase; RB—retinoblastoma protein; ERK—extracellular signal-regulated kinase.

**Table 2:** Currently Approved Tumor-Agnostic Therapies (FDA & EMA).

Drug (INN)	Brand Name	Mechanism/Biomarker Indication	Approval Agencies (Tumor-Agnostic)
**Dabrafenib +** **Trametinib**	Tafinlar + Mekinist	BRAF V600E mutation (BRAF/MEK inhibition)	FDA
**Dostarlimab**	Jemperli	Immune checkpoint inhibitor; dMMR/MSI-H	FDA
**Entrectinib**	Rozlytrek	NTRK-fusion (also ROS1 in NSCLC) inhibitor	FDA, EMA
**Larotrectinib**	Vitrakvi	NTRK-fusion inhibitor	FDA, EMA
**Pembrolizumab**	Keytruda	Immune checkpoint inhibitor; MSI-H/dMMR; TMB-H (TMB ≥10 mutations/Mb)	FDA, EMA
**Repotrectinib**	Augtyro	NTRK-fusion inhibitor	FDA, EMA
**Selpercatinib**	Retsevmo	RET-fusion inhibitor	FDA, EMA
**Trastuzumab deruxtecan**	Enhertu	HER2 overexpression (IHC 3+) ADC	FDA

INN—International Nonproprietary Names.

**Table 3:** FDA-approved targeted therapies for Non-Small Cell Lung Cancer (NSCLC).

Drug (INN)	Brand Name	Primary Target/Mechanism of Action
Adagrasib *	Krazati	KRAS G12C inhibitor
Afatinib *	Giotrif	EGFR inhibitor
Alectinib *	Alecensa	ALK inhibitor
Amivantamab *	Rybrevant	Bispecific antibody (EGFR exon 20 insertion & MET)
Atezolizumab *	Tecentriq	PD-L1 inhibitor
**Atezolizumab + hyaluronidase**	Tecentriq Hybreza	PD-L1 inhibitor (subcutaneous)
Bevacizumab *	Avastin	VEGF monoclonal antibody (anti-angiogenic)
Binimetinib *	Mektovi	MEK inhibitor
Brigatinib *	Alunbrig	ALK inhibitor
Capmatinib *	Tabrecta	MET inhibitor
Cemiplimab *	Libtayo	PD-1 inhibitor
Ceritinib *	Zykadia	ALK inhibitor
Crizotinib *	Xalkori	ALK & ROS1 inhibitor
Dabrafenib *	Tafinlar	BRAF inhibitor (V600E mutation)
Dacomitinib *	Vizimpro	EGFR inhibitor
Durvalumab *	Imfinzi	PD-L1 inhibitor
Encorafenib *	Braftovi	BRAF inhibitor
**Ensartinib**	Ensacove	ALK inhibitor
Entrectinib *	Rozlytrek	NTRK & ROS1 inhibitor
Erlotinib *	Tarceva	EGFR inhibitor
Gefitinib *	Iressa	EGFR inhibitor
Ipilimumab *	Yervoy	CTLA-4 inhibitor
Lazertinib *	Lazcluze	EGFR inhibitor
Lorlatinib *	Lorbrena, Lorviqua	ALK inhibitor
**Necitumumab**	Portrazza	EGFR antibody
Nivolumab *	Opdivo	PD-1 inhibitor
Osimertinib *	Tagrisso	EGFR inhibitor (including T790M mutation)
Pembrolizumab *	Keytruda	PD-1 inhibitor
**Pralsetinib**	Gavreto	RET inhibitor
Ramucirumab *	Cyramza	VEGFR2 antibody (anti-angiogenic)
Repotrectinib *	Augtyro	ROS1 & NTRK inhibitor
Selpercatinib *	Retsevmo	RET inhibitor
Sotorasib *	Lumykras	KRAS G12C inhibitor
Tepotinib *	Tepmetko	MET inhibitor
Trametinib *	Mekinist	MEK inhibitor
Trastuzumab deruxtecan *	Enhertu	HER2-directed ADC
Tremelimumab *	Imjudo	CTLA-4 inhibitor
**Zenocutuzumab**	Bizengri	Bispecific antibody (NRG1 fusion)

INN—International Nonproprietary Names, * Approval by EMA.

**Table 4:** FDA-approved targeted therapies for Small Cell Lung Cancer (SCLC).

Drug (INN)	Brand Name	Primary Target/Mechanism of Action
Atezolizumab *	Tecentriq	PD-L1 inhibitor (first-line with chemotherapy)
Durvalumab *	Imfinzi	PD-L1 inhibitor (first-line with chemotherapy)
**Nivolumab**	Opdivo	PD-1 inhibitor (withdrawn U.S. indication; studied in relapsed SCLC)
**Pembrolizumab**	Keytruda	PD-1 inhibitor (withdrawn U.S. indication; studied in relapsed SCLC)
**Tarlatamab**	Imdelltra	DLL3-targeted bispecific T-cell engager

INN—International Nonproprietary Names, * Approval by EMA.

**Table 5:** FDA-approved targeted therapies for breast cancer.

Drug (INN)	Brand Name	Primary Target/Mechanism of Action
Abemaciclib *	Verzenio	CDK4/6 inhibitor
Alpelisib *	Piqray	PI3K inhibitor
Anastrozole *	Arimidex	Aromatase inhibitor
Capivasertib *	Truqap	AKT inhibitor
Datopotamab deruxtecan *	Datroway	TROP2-directed ADC
Elacestrant *	Orserdu	Selective ER degrader (SERD)
Everolimus *	Afinitor	mTOR inhibitor
**Exemestane**	Aromasin	Aromatase inhibitor
Fulvestrant *	Faslodex	SERD
**Goserelin**	Zoladex	LHRH agonist
Inavolisib *	Itovebi	PI3Kα inhibitor
Lapatinib *	Tykerb, Tyverb	HER2/EGFR TKI
Letrozole *	Femara	Aromatase inhibitor
**Margetuximab**	Margenza	HER2 monoclonal antibody
Neratinib *	Nerlynx	HER2 TKI
Olaparib *	Lynparza	PARP inhibitor
Palbociclib *	Ibrance	CDK4/6 inhibitor
Pembrolizumab *	Keytruda	PD-1 immune checkpoint inhibitor
Pertuzumab *	Perjeta	HER2 monoclonal antibody
**Pertuzumab, trastuzumab, and hyaluronidase**	Phesgo	Subcutaneous HER2 antibody combination
Ribociclib *	Kisqali	CDK4/6 inhibitor
**Ribociclib + letrozole**	Kisqali Femara Co-Pack	CDK4/6 inhibitor + aromatase inhibitor
Sacituzumab govitecan *	Trodelvy	TROP2-directed ADC
Talazoparib *	Talzenna	PARP inhibitor
Tamoxifen *	Soltamox	SERM
Toremifene *	Fareston	SERM
Trastuzumab *	Herceptin	HER2 monoclonal antibody
Trastuzumab deruxtecan *	Enhertu	HER2-directed ADC
Trastuzumab emtansine *	Kadcyla	HER2-directed ADC
**Tucatinib**	Tukysa	HER2 TKI

INN—International Nonproprietary Names, * Approval by EMA.

**Table 6:** FDA-approved targeted therapies for colorectal cancer.

Drug (INN)	Brand Name	Primary Target/Mechanism of Action
**Adagrasib**	Krazati	KRAS G12C inhibitor
Aflibercept *	Zaltrap	VEGF decoy receptor (anti-angiogenic fusion protein)
Bevacizumab *	Avastin	VEGF monoclonal antibody (anti-angiogenic)
Cetuximab *	Erbitux	EGFR monoclonal antibody (RAS wild-type only)
Encorafenib *	Braftovi	BRAF inhibitor (used with cetuximab in BRAF V600E CRC)
Fruquintinib *	Fruzaqla	VEGFR tyrosine kinase inhibitor (anti-angiogenic)
Ipilimumab *	Yervoy	CTLA-4 immune checkpoint inhibitor
Nivolumab *	Opdivo	PD-1 immune checkpoint inhibitor
Panitumumab *	Vectibix	EGFR monoclonal antibody (RAS wild-type only)
Pembrolizumab *	Keytruda	PD-1 immune checkpoint inhibitor
Ramucirumab *	Cyramza	VEGFR2 monoclonal antibody (anti-angiogenic)
Regorafenib *	Stivarga	Multikinase inhibitor (VEGFR, KIT, RET, RAF)
Sotorasib *	Lumykras	KRAS G12C inhibitor
**Tucatinib**	Tukysa	HER2 tyrosine kinase inhibitor

INN—International Nonproprietary Names, * Approval by EMA.

**Table 7:** FDA-approved targeted therapies for prostate cancer.

Drug (INN)	Brand Name(s)	Primary Target/Mechanism of Action
Abiraterone *	Zytiga	CYP17 inhibitor (androgen biosynthesis blockade)
Apalutamide *	Erleada	Androgen receptor (AR) inhibitor
**Bicalutamide**	Casodex	First-generation AR antagonist
Cabazitaxel *	Jevtana	Taxane chemotherapy (microtubule inhibitor)
Darolutamide *	Nubeqa	AR inhibitor
Degarelix *	Firmagon	GnRH antagonist (suppresses testosterone)
Enzalutamide *	Xtandi	AR inhibitor
**Flutamide**	–	First-generation AR antagonist
**Goserelin**	Zoladex	LHRH agonist (medical castration)
**Leuprolide**	Lupron Depot, Eligard	LHRH agonist (medical castration)
Leuprolide *	Camcevi	LHRH agonist (long-acting)
Lutetium (^177^Lu) vipivotide tetraxetan *	Pluvicto	PSMA-targeted radioligand therapy
**Nilutamide**	Nilandron	First-generation AR antagonist
Niraparib + Abiraterone *	Akeega	PARP inhibitor + CYP17 inhibitor
Olaparib *	Lynparza	PARP inhibitor (HRR-mutated prostate cancer)
Radium-223 dichloride (^223^RaCl_2_) *	Xofigo	Alpha-emitting radiopharmaceutical (targets bone metastases)
Relugolix *	Orgovyx	Oral GnRH antagonist
**Rucaparib**	Rubraca	PARP inhibitor (BRCA-mutated prostate cancer)
Talazoparib *	Talzenna	PARP inhibitor (HRR-mutated prostate cancer)
**Triptorelin**	Trelstar	LHRH agonist (medical castration)

INN—International Nonproprietary Names, * Approval by EMA.

**Table 8:** FDA-approved targeted therapies for stomach (gastric) cancer.

Drug (INN)	Brand Name	Primary Target/Mechanism of Action
Nivolumab *	Opdivo	PD-1 immune checkpoint inhibitor
Pembrolizumab *	Keytruda	PD-1 immune checkpoint inhibitor
Ramucirumab *	Cyramza	VEGFR2 monoclonal antibody (anti-angiogenic)
Tislelizumab *	Tevimbra	PD-1 immune checkpoint inhibitor
Trastuzumab *	Herceptin	HER2 monoclonal antibody
Trastuzumab deruxtecan *	Enhertu	HER2-directed antibody–drug conjugate
Zolbetuximab *	Vyloy	Claudin 18.2 monoclonal antibody

INN—International Nonproprietary Names, * Approval by EMA.

**Table 9:** FDA-approved targeted therapies for liver and bile duct cancer.

Drug (INN)	Brand Name	Primary Target/Mechanism of Action
Atezolizumab *	Tecentriq	PD-L1 immune checkpoint inhibitor
**Atezolizumab + hyaluronidase**	Tecentriq Hybreza	PD-L1 inhibitor (subcutaneous formulation)
**Bevacizumab**	Avastin	VEGF monoclonal antibody (anti-angiogenic)
Cabozantinib *	Cabometyx	Multikinase inhibitor (VEGFR, MET, AXL)
Durvalumab *	Imfinzi	PD-L1 immune checkpoint inhibitor
Futibatinib *	Lytgobi	FGFR2 inhibitor (cholangiocarcinoma)
Ipilimumab *	Yervoy	CTLA-4 immune checkpoint inhibitor
Ivosidenib *	Tibsovo	IDH1 inhibitor (cholangiocarcinoma)
Lenvatinib *	Lenvima	Multikinase inhibitor (VEGFR, FGFR, RET, KIT)
Nivolumab *	Opdivo	PD-1 immune checkpoint inhibitor
Pembrolizumab *	Keytruda	PD-1 immune checkpoint inhibitor
Pemigatinib *	Pemazyre	FGFR2 inhibitor (cholangiocarcinoma)
Ramucirumab *	Cyramza	VEGFR2 monoclonal antibody
Regorafenib *	Stivarga	Multikinase inhibitor (VEGFR, KIT, RET, RAF)
Sorafenib *	Nexavar	Multikinase inhibitor (VEGFR, RAF, KIT)
Tremelimumab *	Imjudo	CTLA-4 immune checkpoint inhibitor
Zanidatamab *	Ziihera	HER2 bispecific antibody (HER2-amplified biliary cancer)

INN—International Nonproprietary Names, * Approval by EMA.

**Table 10:** FDA-approved targeted therapies for thyroid cancer.

Drug (INN)	Brand Name	Primary Target/Mechanism of Action
Cabozantinib *	Cometriq	Multikinase inhibitor (VEGFR, RET, MET)
**Dabrafenib**	Tafinlar	BRAF V600E inhibitor (used with trametinib)
Lenvatinib *	Lenvima	Multikinase inhibitor (VEGFR, FGFR, RET, KIT)
**Pralsetinib**	Gavreto	RET-selective inhibitor
Selpercatinib *	Retsevmo	RET-selective inhibitor
Sorafenib *	Nexavar	Multikinase inhibitor (VEGFR, RAF, KIT)
**Trametinib**	Mekinist	MEK1/2 inhibitor (used with dabrafenib)
Vandetanib *	Caprelsa	Multikinase inhibitor (VEGFR, EGFR, RET)

INN—International Nonproprietary Names, * Approval by EMA.

**Table 11:** FDA-approved targeted therapies for bladder cancer.

Drug (INN)	Brand Name	Primary Target/Mechanism of Action
Atezolizumab *	Tecentriq	PD-L1 inhibitor (immune checkpoint blockade)
Avelumab *	Bavencio	PD-L1 inhibitor; also approved as maintenance therapy
Enfortumab vedotin *	Padcev	Nectin-4-directed ADC
Erdafitinib *	Balversa	FGFR2/3 inhibitor
Nivolumab *	Opdivo	PD-1 inhibitor (immune checkpoint blockade)
**Nogapendekin alfa inbakicept**	Anktiva	IL-15 receptor agonist (immune system activation)
Pembrolizumab *	Keytruda	PD-1 inhibitor (immune checkpoint blockade)

INN—International Nonproprietary Names, * Approval by EMA.

**Table 12:** FDA-approved targeted therapies for RCC.

Drug (INN)	Brand Name	Primary Target/Mechanism of Action
Avelumab *	Bavencio	PD-L1 inhibitor (immune checkpoint blockade)
Axitinib *	Inlyta	VEGFR tyrosine kinase inhibitor
Belzutifan *	Welireg	HIF-2α inhibitor (VHL-associated RCC and advanced RCC)
Bevacizumab *	Avastin	VEGF monoclonal antibody (anti-angiogenic)
Cabozantinib *	Cabometyx	Multikinase inhibitor (VEGFR, MET, AXL)
Everolimus *	Afinitor	mTOR inhibitor
Ipilimumab *	Yervoy	CTLA-4 immune checkpoint inhibitor
Lenvatinib *	Lenvima, Kisplyx	Multikinase inhibitor (VEGFR, FGFR, RET, KIT)
Nivolumab *	Opdivo	PD-1 immune checkpoint inhibitor
Pazopanib *	Votrient	VEGFR tyrosine kinase inhibitor
Pembrolizumab *	Keytruda	PD-1 immune checkpoint inhibitor
Sorafenib *	Nexavar	Multikinase inhibitor (VEGFR, RAF, KIT)
Sunitinib *	Sutent	VEGFR/PDGFR tyrosine kinase inhibitor
Temsirolimus *	Torisel	mTOR inhibitor
Tivozanib *	Fotivda	VEGFR tyrosine kinase inhibitor

INN—International Nonproprietary Names, * Approval by EMA.

**Table 13:** FDA-approved targeted therapies for leukemias.

Drug (INN)	Brand Name	Primary Target/Mechanism of Action
Acalabrutinib *	Calquence	BTK inhibitor (CLL)
**Alemtuzumab**	Campath	CD52 monoclonal antibody (CLL)
Asciminib *	Scemblix	BCR-ABL allosteric inhibitor (CML)
Avapritinib *	Ayvakit	PDGFRA/PDGFRα inhibitor (rare leukemias)
Blinatumomab *	Blincyto	CD19/CD3 bispecific T-cell engager (ALL)
Bosutinib *	Bosulif	BCR-ABL TKI (CML)
Brexucabtagene autoleucel *	Tecartus	CAR-T cell therapy, CD19-directed (ALL)
Dasatinib *	Sprycel	BCR-ABL TKI (CML, ALL)
Duvelisib *	Copiktra	PI3Kδ inhibitor (CLL/SLL)
**Enasidenib**	Idhifa	IDH2 inhibitor (AML)
Gemtuzumab ozogamicin *	Mylotarg	CD33-directed ADC (AML)
Gilteritinib *	Xospata	FLT3 inhibitor (AML)
Glasdegib *	Daurismo	Hedgehog pathway inhibitor (AML)
Ibrutinib *	Imbruvica	BTK inhibitor (CLL, ALL)
Idelalisib *	Zydelig	PI3Kδ inhibitor (CLL)
Imatinib *	Gleevec, Glivec	BCR-ABL TKI (CML, ALL)
Inotuzumab ozogamicin *	Besponsa	CD22-directed ADC (ALL)
Ivosidenib *	Tibsovo	IDH1 inhibitor (AML)
Lisocabtagene maraleucel *	Breyanzi	CAR-T cell therapy, CD19-directed
Midostaurin *	Rydapt	FLT3 inhibitor (AML)
Nilotinib *	Tasigna	BCR-ABL TKI (CML)
Obecabtagene autoleucel *	Aucatzyl	CAR-T cell therapy (CD19)
Obinutuzumab *	Gazyva	CD20 monoclonal antibody (CLL)
Ofatumumab *	Arzerra	CD20 monoclonal antibody (CLL)
**Olutasidenib**	Rezlidhia	IDH1 inhibitor (AML)
Pemigatinib *	Pemazyre	FGFR2/FGFR1 inhibitor (rare leukemias)
Pirtobrutinib *	Jaypirca	Non-covalent BTK inhibitor (CLL)
Ponatinib *	Iclusig	BCR-ABL TKI (esp. T315I-mutated CML)
Quizartinib *	Vanflyta	FLT3 inhibitor (AML)
**Revumenib citrate**	Revuforj	Menin inhibitor (KMT2A/NPM1-mutant leukemias)
Rituximab *	Rituxan, Rixathon	CD20 monoclonal antibody (CLL)
**Rituximab + hyaluronidase**	Rituxan Hycela	Subcutaneous CD20 monoclonal antibody
Tagraxofusp *	Elzonris	CD123-directed fusion protein
Tisagenlecleucel *	Kymriah	CAR-T cell therapy, CD19-directed (ALL)
**Tretinoin**	Vesanoid	Induces differentiation (APL)
Venetoclax *	Venclexta	BCL-2 inhibitor (CLL, AML)
Zanubrutinib *	Brukinsa	BTK inhibitor (CLL)

INN—International Nonproprietary Names, * Approval by EMA.

**Table 14:** FDA-approved targeted therapies for pancreatic cancer.

Drug (INN)	Brand Name	Primary Target/Mechanism of Action
Belzutifan *	Welireg	HIF-2α inhibitor (VHL-associated tumors)
Erlotinib *	Tarceva	EGFR tyrosine kinase inhibitor
Everolimus *	Afinitor	mTOR inhibitor (pancreatic neuroendocrine tumors)
Olaparib *	Lynparza	PARP inhibitor (BRCA1/2-mutated PDAC)
Sunitinib *	Sutent	Multikinase inhibitor (VEGFR, PDGFR, KIT; pNETs)
**Zenocutuzumab**	Bizengri	HER2/HER3 bispecific antibody (NRG1 fusions)

INN—International Nonproprietary Names, * Approval by EMA.

**Table 15:** FDA-approved targeted therapies for brain tumors.

Drug (INN)	Brand Name	Primary Target/Mechanism of Action
Belzutifan *	Welireg	HIF-2α inhibitor (Von Hippel–Lindau-associated tumors)
**Bevacizumab**	Avastin	VEGF monoclonal antibody (anti-angiogenic)
Dabrafenib *	Finlee, Tafinlar	BRAF inhibitor (V600E mutation)
Everolimus *	Votubia, Afinitor	mTOR inhibitor (TSC-associated SEGA)
**Tovorafenib**	Ojemda	Pan-RAF inhibitor (BRAF-altered pediatric low-grade glioma)
Trametinib *	Spexotras, Mekinist	MEK inhibitor (MAPK pathway)
Vorasidenib *	Voranigo	Mutant IDH1/2 inhibitor (IDH-mutant low-grade glioma)

INN—International Nonproprietary Names, * Approval by EMA.

**Table 16:** FDA-approved targeted therapies for skin cancer.

Drug (INN)	Brand Name	Primary Target/Mechanism of Action
Alitretinoin *	Panretin	Retinoid (RXR/RAR agonist, cutaneous T-cell lymphoma)
**Atezolizumab**	Tecentriq	PD-L1 inhibitor (Merkel cell carcinoma, melanoma)
**Atezolizumab + hyaluronidase**	Tecentriq Hybreza	PD-L1 inhibitor (subcutaneous formulation)
Avelumab *	Bavencio	PD-L1 inhibitor (Merkel cell carcinoma)
Binimetinib *	Mektovi	MEK inhibitor (BRAF-mutant melanoma, with encorafenib)
Cemiplimab *	Libtayo	PD-1 inhibitor (cSCC, melanoma)
Cobimetinib *	Cotellic	MEK inhibitor (with vemurafenib in BRAF+ melanoma)
**Cosibelimab**	Unloxcyt	PD-1 inhibitor (cSCC, melanoma)
Dabrafenib *	Tafinlar	BRAF V600E/K inhibitor (melanoma, with trametinib)
Encorafenib *	Braftovi	BRAF V600E/K inhibitor (melanoma, with binimetinib)
Ipilimumab *	Yervoy	CTLA-4 inhibitor (melanoma, in combination regimens)
Nivolumab *	Opdivo	PD-1 inhibitor (melanoma, uveal melanoma combinations)
Nivolumab + relatlimab *	Opdualag	PD-1 + LAG-3 inhibition (melanoma)
Pembrolizumab *	Keytruda	PD-1 inhibitor (melanoma, cSCC, Merkel cell carcinoma)
Retifanlimab *	Zynyz	PD-1 inhibitor (Merkel cell carcinoma, cSCC)
Sonidegib *	Odomzo	Hedgehog pathway (SMO) inhibitor (BCC)
Tebentafusp *	Kimmtrak	Bispecific gp100/HLA-A *02:01/CD3 engager (uveal melanoma)
Trametinib *	Mekinist	MEK inhibitor (with dabrafenib in BRAF+ melanoma)
Vemurafenib *	Zelboraf	BRAF V600E inhibitor (melanoma, with cobimetinib)
Vismodegib *	Erivedge	Hedgehog pathway (SMO) inhibitor (BCC)

INN—International Nonproprietary Names, * Approval by EMA.

**Table 17:** FDA-approved targeted therapies for ovarian epithelial, fallopian tube, and primary peritoneal cancers.

Drug (INN)	Brand Name	Primary Target/Mechanism of Action
Bevacizumab *	Avastin	VEGF monoclonal antibody (anti-angiogenic)
Mirvetuximab soravtansine *	Elahere	FRα-directed antibody–drug conjugate
Niraparib *	Zejula	PARP inhibitor
Olaparib *	Lynparza	PARP inhibitor
Rucaparib *	Rubraca	PARP inhibitor

INN—International Nonproprietary Names, * Approval by EMA.

## Data Availability

No original data was generated in this article.
